# Highly sensitive voltammetric determination of the fungicide fenhexamid using a cost-effective and disposable pencil graphite electrode

**DOI:** 10.1007/s00604-024-06804-4

**Published:** 2024-11-29

**Authors:** Teslime Erşan, Didem Giray Dilgin, Ayhan Oral, Sławomira Skrzypek, Mariola Brycht, Yusuf Dilgin

**Affiliations:** 1 Department of Chemistry, Faculty of Science, Çanakkale Onsekiz Mart University, Çanakkale, 17020 Türkiye; 2 Secondary Science and Mathematics Education Department, Faculty of Education, Çanakkale Onsekiz Mart University, Çanakkale, 17100 Türkiye; 3 University of Lodz, Faculty of Chemistry, Department of Inorganic and Analytical Chemistry, Tamka 12, 91-403 Lodz, Poland

**Keywords:** Pesticide detection, Electrochemical sensor, Graphite electrode, Differential pulse voltammetry, Electroanalysis

## Abstract

A differential pulse voltammetric (DPV) method is proposed for the highly sensitive determination of fenhexamid (FHX) based on both electrooxidation and electroreduction processes using a disposable and cost-effective pencil graphite electrode (PGE). The electrochemical oxidation and reduction mechanisms of FHX at the PGE were elucidated by recording cyclic voltammograms at various pH values of Britton–Robinson buffer (BRB) solutions at a scan rate of 50 mV s^−1^ and different scan rate values in the range 10–400 mV s^−1^ at selected pH of BRB (pH 2.0). Differential pulse voltammograms recorded under optimized conditions revealed an oxidation peak of FHX around + 0.65 V and a reduction peak of FHX around + 0.45 V. The DPV analysis of FHX revealed two linear ranges: 0.001–0.01 µmol L^−1^ and 0.01–5.0 µmol L^−1^ for the anodic peak, and 0.001–0.1 µmol L^−1^ and 0.1–5.0 µmol L^−1^ for the cathodic peak. The limits of detection were 0.34 nmol L^−1^ and 0.32 nmol L^−1^ for the anodic and cathodic peaks, respectively. The proposed methodology demonstrated satisfactory selectivity, as selected pesticides, certain electroactive compounds, and cationic species tested did not interfere with the voltammetric determination of FHX, particularly during its reduction. The recovery results, showing values close to 100% obtained from the analysis of real samples spiked with FHX, indicated that this methodology can accurately determine FHX in water and soil samples.

**Graphical abstract:**

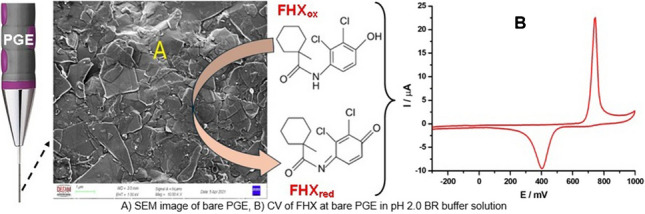

**Supplementary Information:**

The online version contains supplementary material available at 10.1007/s00604-024-06804-4.

## Introduction

Pesticides are extensively used in modern agriculture to safeguard fruits and vegetables from diseases caused by fungi, weeds, and insects, ultimately enhancing crop yields. Among these pesticides, fungicides play a crucial role in agriculture by controlling plant-damaging fungi, including leaf rusts, gray molds, mildews, blasts (particularly in rice), blights, and anthracnose [1]. Fungicides protect target plants by either killing fungi and their spores or inhibiting their growth. *Botrytis cinerea*, a common pathogenic fungus, is a major cause of gray mold disease in various crops and plants [2, 3]. This disease significantly reduces the quality and yield of fruits, vegetables, and ornamental plants, with grapevines being particularly vulnerable [2, 3].

Fenhexamid (FHX), a hydroxyanilide derivative, is a prominent fungicide widely employed to combat fungal diseases in fruits and vegetables [2]. FHX exhibits potent preventive activity against *Botrytis cinerea* and other pathogenic fungi such as *Sclerotinia sclerotiorum* and *Monilinia* spp*. *[3, 4]*.* Additionally, FHX has been utilized to induce resistance in transgenic *Magnaporthe oryzae* by targeting the FfERG27 gene of *Fusarium fujikuroi* [5]*.* The impact of FHX on *Botrytis cinerea* is demonstrated by its strong inhibitory effect on germ tube elongation and mycelium growth during the developmental stages following germination. Moreover, FHX inhibits germination when applied to *Botrytis cinerea* conidia at relatively higher concentrations. Shortly after germination begins, germ tubes from spores exposed to FHX stop growing, and granular structures form within the cytoplasm [4]. This is followed by coagulation and shrinkage, ultimately leading to the collapse and death of spores and germ tubes before they can penetrate the plant surface. Studies have revealed that FHX inhibits fungal diseases by blocking sterol 3-ketoreductase, which leads to the inhibition of ergosterol C4-demethylation [3–6]. Antifungals used in both medical and agricultural sectors target specific enzymes in the ergosterol biosynthesis pathway, such as 14α-demethylase, squalene epoxidase, Δ14-reductase, Δ7-Δ8-isomerase, and 3-ketoreductase, all of which are involved in C4-demethylation [6]. Inhibitors of C4-demethylation, such as FHX, is the most commonly used antifungals in agriculture. As a hydroxyanilide, FHX specifically targets the 3-ketoreductase enzyme in the C-4-demethylating complex, effectively inhibiting the gray mold pathogen *Botrytis cinerea* and other related species, such as *Sclerotinia* spp. and *Monilinia* spp. [6]. Thus, FHX plays a crucial role in combating fungal diseases and holds significant importance as an antifungal agent in agriculture.

The widespread use of pesticides in agriculture and related food sectors leads to their easy leaching into the environment and subsequent degradation into different forms [7]. Like other toxic pesticides, FHX can enter natural aquatic systems through multiple pathways, such as uncontrolled use during production, accidental introduction, aerial spray drift during application, and runoff from vineyards into surface water [8]. As a result, fungicides like FHX have the potential to persist in the environment, even at low concentrations. This makes soil and aquatic ecosystems vulnerable to contamination, posing a potential risk to human and animal health. Therefore, extensive research is crucial to fully understand the environmental and health impacts of FHX. The impact of FHX on human health is significant due to the homology between the target enzymes, such as 3-ketoreductase and C4-demethylase, and similar systems in the human body [9]. For instance, 3-ketoreductase and 14α-demethylase, primarily encoded by the erg27 and erg11 genes in fungi, correspond to the human genes HSD17B7 and CYP51A1, respectively [10, 11]. In this context, the interaction of FHX with calf thymus DNA was studied, and it was reported that FHX exhibits low genetic toxicity, as it induces only slight changes in the DNA conformation and has low affinity for binding to DNA [9]. In another study, the effect of FHX and myclobutanil, both individually and in combination, was tested on two human cell lines (SH-SY5Y neuronal cells and U-251 MG glial cells). No significant differences were found in cell viability or mitochondrial membrane potential between the two cell lines when exposed to these agents. However, the study reported that: (i) SH-SY5Y cells exhibited significantly higher susceptibility to oxidative stress, as indicated by greater depletion of total thiols compared to astrocytic cells, and (ii) treatment with both compounds resulted in notable changes in the expression of several genes involved in cell cycle control, growth, DNA repair, and apoptosis regulation [12]. Studies on breast cancer models suggest that FHX, while relatively safe as a fungicide, can promote breast cancer cell growth via the estrogen receptor and/or phosphatidylinositol 3-kinase pathways, potentially acting as an endocrine-disrupting chemical [2, 13, 14]. In another study, FHX was found to inhibit the growth of *Chlorella vulgaris*, a common green alga in aquatic environments, disrupting its antioxidant production and cellular structure [15]. Additionally, the effects of FHX and atrazine on the growth and oxidative stress of *Scenedesmus obliquus* (a type of microalgae) were investigated, and it was reported that changes in antioxidant enzyme activities due to fungicide treatment showed an antagonistic interaction in algae after exposure [16]. Similarly, FHX exposure to *Tubifex tubifex* (a type of oligochaete) led to decreased growth, reduced protein and glycogen levels, toxic effects, and increased oxidative stress, as observed through changes in antioxidative enzyme activities [8]. To enhance the efficacy of fungicides and reduce their adverse environmental and health impacts, recent efforts have focused on carrier-free, self-assembled nanoparticles (NPs). For instance, combining FHX with prochloraz (PRO) and polyhexamethylene biguanide (PHMB) resulted in a synergistic effect on fungicidal and antimicrobial activity [17, 18]. FHX-PRO [17] or FHX-PHMB [18] NPs demonstated higher efficacy against fungi and bacteria compared to the use of FHX alone. Genotoxicity studies of these NPs showed that they did not further increase the cytotoxicity of FHX. Despite these efforts to mitigate its environmental and health effects, the widespread use and release of FHX remain a significant concern. FHX is considered a pollutant that can adversely affect human and animal health, as well as soil and water systems, due to its accumulation in food production and its easy release into the environment. Given these concerns, accurate determination of FHX levels is essential to ensure food quality, protect human health, and preserve environmental integrity.

Several analytical methods have been developed to determine FHX residues in various samples, including a gold nanoparticle–based immunochromatographic assay using liquid chromatography (LC) coupled with mass spectrometry (MS) [19], gas chromatography with nitrogen phosphorous and electron capture detectors [20, 21], high-performance liquid chromatography with UV detectors [21, 22], LC-electrospray ionization or atmospheric pressure chemical ionization-tandem MS/MS [22, 23], and enzyme-linked immunosorbent assay (ELISA) [24–26]. While chromatographic methods offer high selectivity, they have drawbacks such as expensive equipment, the need for highly trained personnel, extensive use of organic solvents, and lengthy sample preparation [19]. ELISA also faces limitations in terms of analysis time. Electrochemical methods, on the other hand, present an attractive alternative due to their simplicity, cost-effectiveness, and high sensitivity. Recently, sensitive and selective electrochemical determination of FHX has been achieved using various carbon-based electrodes such as glassy carbon (GCE), carbon paste (CPE), CPE modified with multi-walled carbon nanotubes, screen-printed carbon, glassy carbon paste (GCPE) [27], and anodically pretreated boron-doped diamond (APT-BDDE) [28]. However, these carbon-based electrodes reported for the determination of FHX [27, 28] are relatively expensive and require a surface pretreatment step. Additionally, they have been used only for electrochemical oxidation of FHX.

In this study, a disposable and cost-effective pencil graphite electrode (PGE) was utilized for the determination of FHX. Our recent studies have demonstrated that the PGE exhibits excellent analytical performance in electrochemical analysis due to its disposability, high sensitivity, mechanical rigidity, renewability, ease of modification, and low cost when compared to other carbon-based electrodes [29–31]. Based on our review of the literature, no previous research has focused on the determination of FHX using the PGE. This study represents the first attempt to determine FHX at the PGE using differential pulse voltammetry (DPV), exploring both its oxidation and reduction processes. This research aims to fill the gap in the existing literature and contribute to the development of efficient and accessible methods for FHX analysis.

## Materials and method

### Chemicals and apparatus

Fenhexamid (FHX, C_14_H_17_Cl_2_NO_2_, purity ≥ 98.0%) was supplied by Merck. The stock FHX solution (5.0 mmol L^−1^) was freshly prepared in absolute methanol, and diluted standard solutions were prepared from this stock solution. Mineral oil (density of 0.862 g mL^−1^), graphite powder (particle size < 50 µm), methanol, K_4_Fe(CN)_6_ × 3H_2_O, K_3_Fe(CN)_6_, H_3_PO_4_, H_2_SO_4_, NaOH, H_3_BO_3_, and interfering compounds (CaCl_2_, MgCl_2_, CuCl_2_, MnSO_4_ × H_2_O, Co(NO_3_)_2_ ZnCl_2_, KCl, NaCl, atrazine, monolinuron, carbendazim, and trifluralin) used in this study were purchased from Merck. An equimolar (1:1) (5.0 mmol L^−1^) K_4_Fe(CN)_6_ × 3H_2_O and K_3_Fe(CN)_6_ redox marker solution was prepared in 0.1 mol L^−1^ KCl. All aqueous solutions were prepared using ultrapure water with a resistivity of 18.2 MΩ cm^−1^ produced by an Elga Option Q7B water purification system. Britton–Robinson buffer (BRB) solutions with various pH values were prepared by mixing known amounts of acids (0.04 mol L^−1^ H_3_PO_4_, 0.04 mol L^−1^ H_3_BO_3_, and 0.04 mol L^−1^ CH_3_COOH containing 0.1 mol L^−1^ KCl) and base (0.2 mol L^−1^ NaOH containing 0.1 mol L^−1^ KCl) solutions.

Electrochemical measurements were undertaken using a CompactStat.h potentiostat (Ivium Technologies, the Netherlands) under the control of IviumSoft (version 1.9) software. Pencil leads (2B, TOMBOW, Japan, Ø: 0.5 mm), GCE (BASi, the USA, Ø: 7 mm), screen-printed carbon electrode (SPCE, Dropsens, Spain, Ø: 4 mm), and CPE (Ø: 3 mm) prepared by mixing of 70% graphite and 30% mineral oil were used as working electrodes. Pencil graphite leads were inserted into a mechanical pencil holder. Platinum wire (BASi, the USA) and AgǀAgClǀKCl_(sat.)_ were used as counter and reference electrodes, respectively. The pH of the BRB solutions was measured using a Hanna Instruments model HI-221 pH meter.

### Electrochemical procedures

The electrochemical behavior of 27.5 µmol L^−1^ FHX was investigated by recording cyclic voltammograms (CVs) using a potential window ranging from − 300 to + 1000 mV with a starting potential of − 300 mV in 0.1 mol L^−1^ H_2_SO_4_ solution and BRB solutions containing 0.1 mol L^−1^ KCl solution, across pH values ranging from 2.0 to 10.0, at a scan rate of 50 mV s^−1^. Additionally, CVs of FHX were recorded at different scan rate values ranging from 10 to 400 mV s^−1^ in a pH 2.0 of BRB solution containing 0.1 mol L^−1^ KCl solution.

The electroanalytical determination of FHX was conducted using DPV. Differential pulse voltammograms (DPVs) of 0.25 µmol L^−1^ FHX were recorded while varying the pH value of the BRB solutions containing 0.1 mol L^−1^ KCl solution, applying potential scanning in both anodic (from 0 to + 1000 mV) and cathodic (from + 1000 to 0 mV) directions using the PGE. Parameters in DPV mode, such as pulse amplitude (ΔE_p_), pulse time (t_p_), step potential (ΔE_s_), and scan rate (ʋ), were optimized by recording DPVs of 0.25 µmol L^−1^ FHX for both its oxidation and reduction in a pH 2.0 of BRB solution containing 0.1 mol L^−1^ KCl solution.

### Real sample analysis

Two water samples (tap water and agricultural irrigation water) and soil samples were utilized to apply the proposed method to real samples.

The tap water was taken from the research laboratory at Çanakkale Onsekiz Mart University (Turkey), while the agricultural irrigation water was collected from the irrigation canal passing through Kangırlı Village in Çanakkale (Turkey). The water samples were spiked with a FHX stock solution (1.0 mmol L^−1^) to obtain spiked water samples containing FHX at three different concentrations (5, 10, and 15 µmol L^−1^). After spiking, the samples were filtered through a 0.22-µm membrane before use.

The soil sample was obtained from an agricultural field in Kangırlı Village (Turkey). A 20 mL of pure methanol (99.8%) was added to 2.0 g of the ground soil sample spiked with a FHX stock solution (1.0 mmol L^−1^) at three different concentrations (10, 20, and 40 µmol L^−1^). The obtained mixtures were then shaken at 500 rpm for 30 min and filtrated through a 0.22-µm membrane. Subsequently, the filtrate was centrifuged at 4000 rpm for 15 min to obtain a clear supernatant.

The DP voltammetric determination of FHX in all real samples was carried out using the standard addition method as follows. Five milliliters of supporting electrolyte (BRB solution at pH 2.0 containing 0.1 mol L^−1^ KCl) was added to the electrochemical cell, and a background voltammogram was recorded in both anodic and cathodic directions. Further, an appropriate volume of the spiked sample was added to the cell, and the voltammogram was recorded under optimized conditions in both anodic and cathodic directions. Next, standard additions of FHX stock solution (1.0 mmol L^−1^) were made to the cell at concentrations of 0.5, 1.0, 1.5, and 2.0 µmol L^−1^, and DPVs were recorded in both anodic and cathodic directions. In the final step, the spiked FHX concentration was calculated from the standard addition plot obtained for each tested sample.

## Results and discussion

### Electrochemical performance of working electrodes

**Fig. 1:**
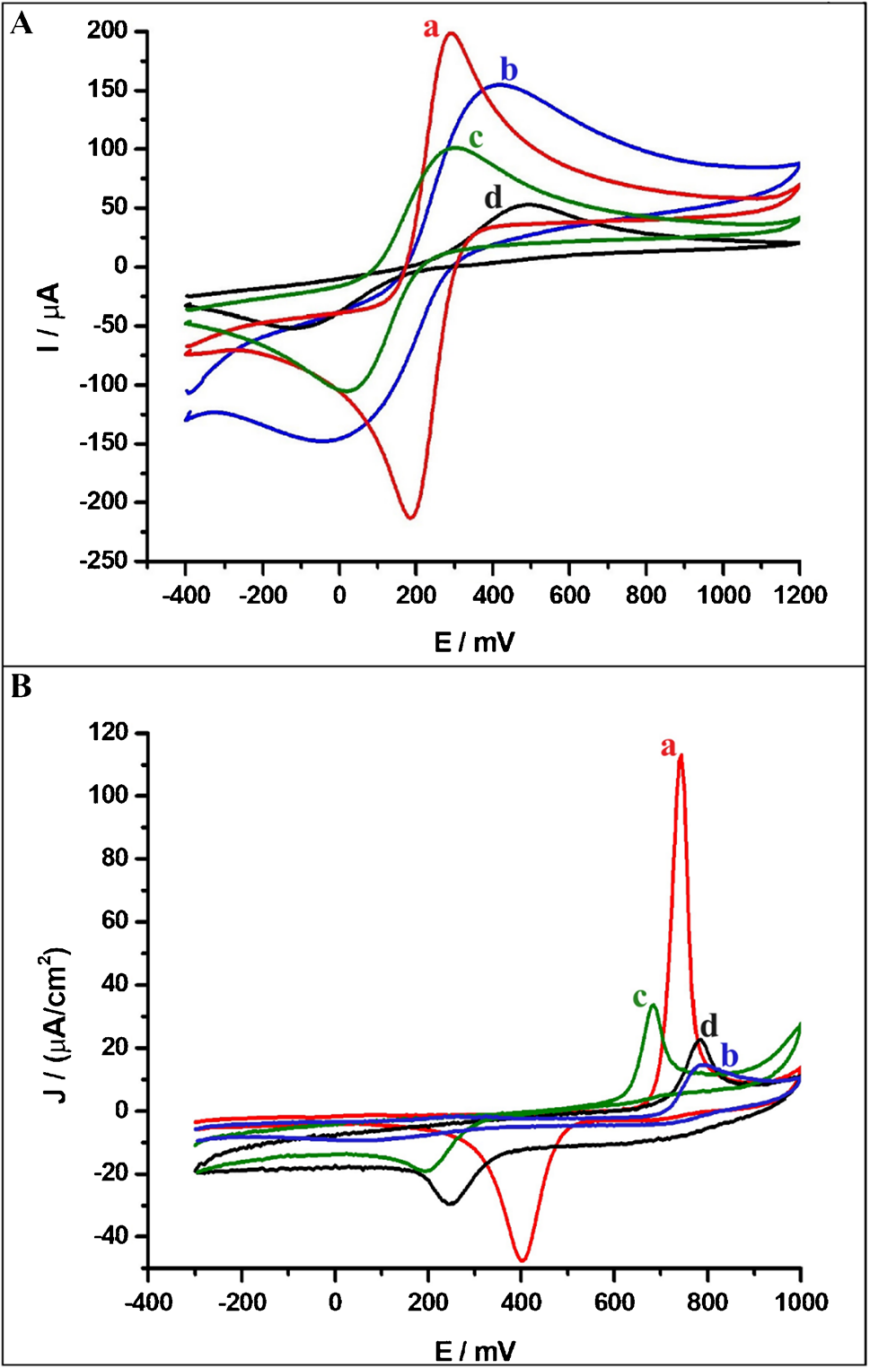
Cyclic voltammograms of (**A**) 5.0 mmol L^−1^ redox marker containing 0.1 mol L^−1^ KCl and (**B**) 27.5 µmol L^−1^ FHX in pH 2.0 of BRB solution containing 0.1 mol L^−1^ KCl solution recorded on the (a) PGE, (b) CPE, (c) SPCE, and (d) GCE at a scan rate of 50 mV s^−1^

To compare the electrochemical performance of working electrodes, i.e., PGE, GCE, SPCE, and CPE, measurements were performed using cyclic voltammetry (CV) with a 5.0 mmol L^**−**1^ [Fe(CN)_6_]^3**−**/4**−**^ redox probe in 0.1 mol L^**−**1^ KCl solution. The CVs of the redox marker on all tested electrodes, recorded with a scan rate of 50 mV s^**−**1^, are presented in Fig. 1A. The results showed that the PGE exhibited better electrochemical performance towards redox marker compared to the other electrodes, as evidenced by the lowest peak potential separation (ΔE_p_) value of 100 mV. Additionally, the anodic and cathodic peak currents of the redox marker further supported this conclusion, with the highest peak currents (I_pa_ of 218 µA and I_pc_ of 215 µA) obtained when using the PGE.

To assess the electroactive surface area (ESA) of the electrodes, CVs of 5.0 mmol L^**−**1^ [Fe(CN)_6_]^3**−**/4**−**^ redox marker were recorded at various scan rates ranging from 50 to 500 mV s^**−**1^ for all electrodes. In all electrodes, [Fe(CN)_6_]^3**−**/4^ exhibited a quasi-reversible redox behavior as the ΔE_p_ values were observed to be higher than 59 mV/n. Therefore, the ESA of the electrodes was estimated using the Randles–Sevcik equation for quasi-reversible systems: *I*_*p*_ = 2.63 × 10^5^ × *n*^3/2^ × ESA × *c*_0_ × *D*^1/2^ × *ʋ*^1/2^ [32–34], where *I*_*p*_, *n*, ESA, *c*_0_, *D*, and *ʋ* represent the peak current (Amper), electron number (*n* = 1), electroactive surface area (cm^2^), concentration of redox probe (5.0 × 10^−6^ mol cm^−3^), diffusion coefficient of the redox probe (6.23 × 10^**−**6^ cm^2^ s^−1^ [32, 33]), and scan rate (V s^**−**1^), respectively). The calculated ESA values and percentage roughness factor (%Rf: (ESA/*A*_geom_) × 100) [34, 35] are given in Table S1. It can be seen that ESA were found to be 0.181 cm^2^ for the PGE, 0.094 cm^2^ for the SPCE, 0.096 cm^2^ for CPE, and 0.050 cm^2^ for the GCE. It is evident that the PGE exhibited the highest ESA, approximately 4.5-times greater than that of the GCE. In addition, the ESA values were found to be larger than the geometric surface area (*A*_geom_) due to the presence of fine surface roughness features in the electrode materials [35]. As shown in Table S1, the highest %Rf values for CPE and PGE indicate that these electrodes exhibit greater surface roughness compared to SPCE and GCE. Moreover, the heterogeneous electron transfers rates (*k*_0_) between the electrodes and the [Fe(CN)_6_]^3**−**/4**−**^ redox probe, as well as the anodic charge transfer coefficient values (α_0_), were calculated by using Nicholson equation *ψ* = 33.47 k_0_ ν^**−**1/2^ and the equation *α*_0_ = *m*_a_/(*m*_a_ + *m*_c_), respectively [36, 37]. Here, ψ is the dimensionless kinetic parameter determined from ΔE_p_, ν is the scan rate, and *m*_a_ and *m*_b_ represent the slopes of the anodic and cathodic peak currents vs. log *ν*, respectively. Obtained results are also given in Table S1.

### Electrochemical behavior of FHX at the PGE

To highlight the superior electrochemical response of FHX on the PGE compared to other carbon-based electrodes, CVs of 27.5 µmol L^−1^ FHX were recorded under the same conditions (supporting electrolyte: BRB solution at pH 2.0 containing 0.1 mol L^−1^ KCl solution and a scan rate of 50 mV s^−1^) using the PGE, GCE, SPCE, and CPE (Fig. 1B). The PGE exhibited exceptional response to the quasi-reversible redox couple of FHX oxidation and reduction compared to other tested electrodes, with sharp anodic and cathodic peaks observed at approximately + 0.75 and + 0.4 V, respectively. The current density (*J*) obtained from the anodic peak of FHX using the PGE was approximately 3.5-, 5.5-, and 8-times greater than those obtained using the SPCE, GCE, and CPE, respectively. Additionally, the *J* value obtained from the cathodic peak at the PGE was 4-, 3-, and 15-times higher those for the SPCE, GCE, and CPE, respectively. The superior electroanalytical activity of the PGE towards FHX can be attributed to its composition of sp^2^-hybridized graphitic carbon and clay minerals, which impart properties such as excellent adsorption capacity, a porous structure, high electrical conductivity, and low background current. The FE-SEM image of the PGE surface (Fig. S1A) reveals that typical segregated platelets are randomly distributed across the surface, forming a layered porous structure of pencil leads. Additionally, the EDX spectrum (Fig. S1B) confirms the composition pencil leads, with weight percentages of 89.07% C, 6.87% O, 3.67% Si, and 0.39% Al, indicating the presence of graphite and aluminosilicate clay minerals. Moreover, the irregular porous surface morphology of the PGE enhances its reactivity and increases the ESA. The edge plane regions of this irregular surface exhibit high resistance to surface passivation, preventing analyte adsorption on the electrode surface and facilitating more efficient electron transfer at the edge plane sites [29, 32, 38]. Consequently, the PGE’s high surface area and abundance of active sites promote rapid electron transfer for FHX detection [38–40].

**Fig. 2:**
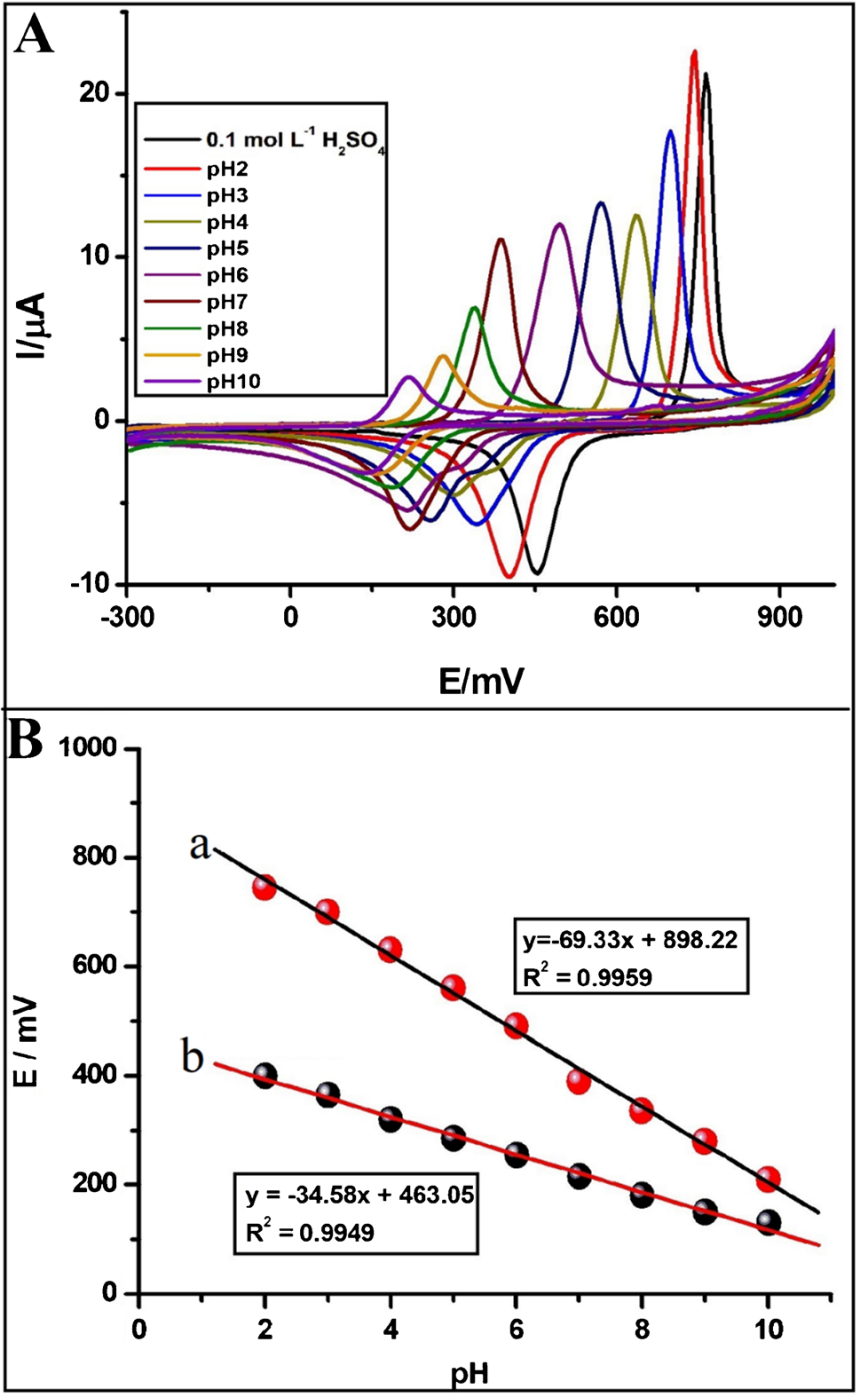
**A** Cyclic voltammograms of 27.5 µmol L^−1^ FHX recorded at the PGE in 0.1 mol L^−1^ H_2_SO_4_ and BRB solutions containing 0.1 mol L^−1^ KCl solution prepared at varying pH values between 2.0 and 10.0 at a scan rate of 50 mV s^−1^. **B** The linear dependences of (a) oxidation and (b) reduction peak potentials vs. pH

**Scheme 1:**
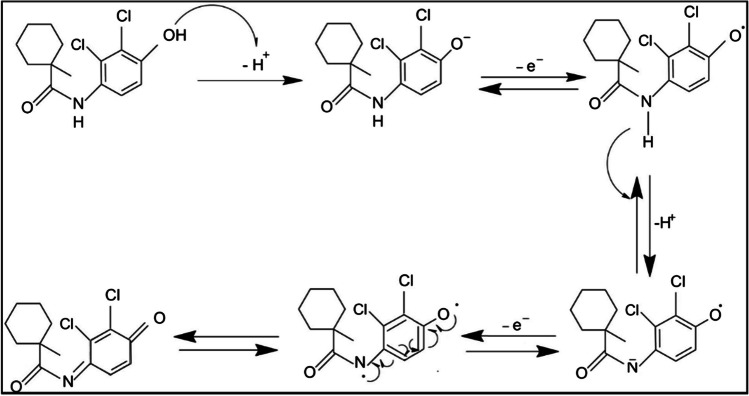
Proposed mechanism of electrochemical behavior of FHX exhibiting a quasi-reversible redox couple at the PGE

To evaluate the effect of pH on the current and potential of both peaks observed at the PGE, CVs of 27.5 µmol L^−1^ FHX were recorded in 0.1 mol L^−1^ H_2_SO_4_ and BRB solutions containing 0.1 mol L^−1^ KCl solution prepared at varying pH values between 2.0 and 10.0 at a scan rate of 50 mV s^−1^. As depicted in Fig. 2A, the peak potentials of the quasi-reversible redox couple of FHX shift in a more negative direction with increasing the basicity of BRB solutions. This shift in peak potentials with changing pH suggests that proton-coupled electron transfer reactions occur during the electrochemical oxidation and reduction of FHX at the PGE [41]. The linear relationship between anodic peak potential (E_pa_) and pH depicted in Fig. 2B is described by the equation: E_pa_ (mV) = − 69.33 pH + 898.2 (*R*^2^ = 0.9959). The slope of this equation (− 69.33 mV pH^−1^) closely aligns with the theoretical Nernstian value of 59 mV pH^−1^, indicating that the redox reaction of FHX on the PGE involves an equal number of protons and electrons. In contrast, the linear equation for cathodic peak potential (E_pc_) vs. pH is described by the equation: E_pc_ = − 34.58 pH + 463.1 (*R*^2^ = 0.9949), where the slope is half the value of the theoretical Nernstian value. Therefore, it can be deduced that the reduction of FHX involves a mechanism with two electrons and one proton. Based on these results, the following mechanism can be proposed, considering that FHX is oxidized by losing two electrons and two protons, and then the oxidized form gains two electrons and one proton during reduction. As depicted in Scheme 1, the hydroxyphenyl group is initially oxidized to a phenoxy radical, which then converts to a quinone form by accepting a proton from the carboxamide group while releasing one electron and one proton from the N–H group. This mechanism has been previously reported by Brycht et al. for the oxidation of FHX at the GCPE [27] and APT-BDDE [28]. Additionally, a similar mechanism has been proposed for the oxidation of amodiaquine, which has a similar structure to FHX [29]. As observed from the proposed mechanism, two radicals are coupled, leading to the formation of a double bond. However, during the reduction of the quinone group, only two electrons and one proton are transferred, resulting in FHX remaining in the phenolate form.

Additionally, the bar graphs illustrating the peak current vs. pH (Fig. S2) demonstrate that both anodic and cathodic peak currents decrease with increasing pH values of the supporting electrolyte. The highest peak current values were obtained at pH 2.0 of BRB solution, which was selected as the optimal medium for further measurements. As the pH values increase, the peak currents decrease, while ΔE_peak_ values decrease and the reversibility increases. This phenomenon can be attributed to the transformation of the hydroxyphenyl group into the phenolate anion in a basic environment, which hinders electron transfer.

**Fig. 3:**
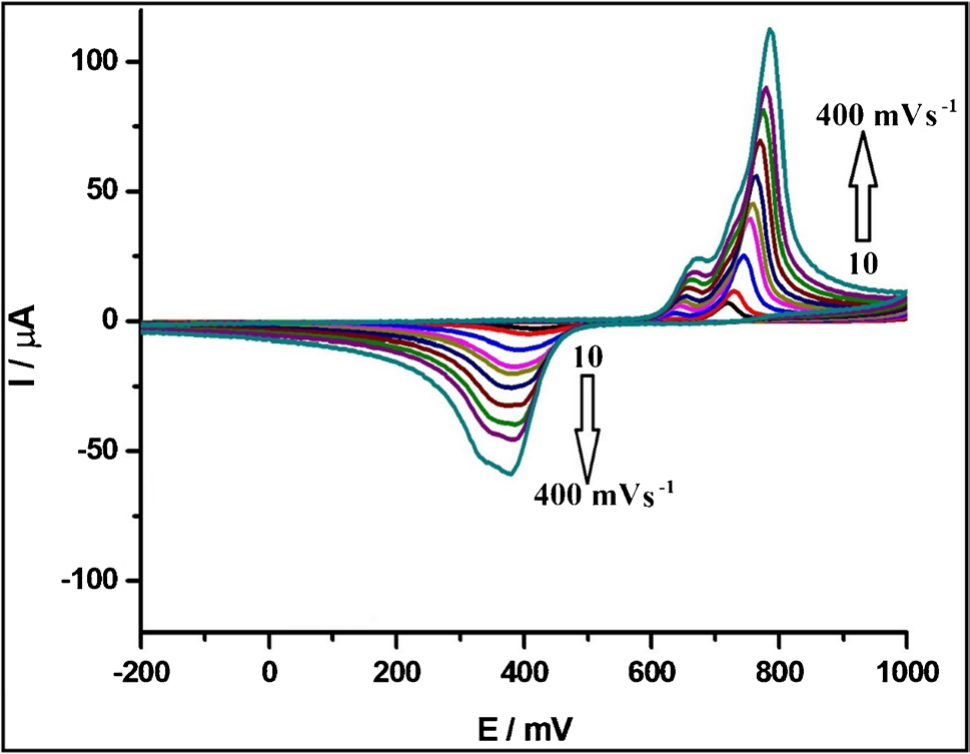
Cyclic voltammograms of 27.5 µmol L^−1^ FHX recorded at the PGE in a pH 2.0 of BRB solution containing 0.1 mol L^−1^ KCl solution at varying scan rates ranging from 10 to 400 mV s^−1^

The effect of scan rate on the peak currents of FHX was examined by recording CVs of 27.5 µmol L^−1^ FHX in a pH 2.0 of BRB solution containing 0.1 mol L^−1^ KCl solution at varying scan rates between 10 and 400 mV s^−1^ (Fig. 3). The resulting CVs indicated that peak currents increase as the scan rate increase. The relationships between peak currents and the square root of the scan rate are shown in the Fig. S3 for both the anodic and cathodic peaks. Notably, the peak currents demonstrated a linear increase with the square root of the scan rate, suggesting a diffusion-controlled process for the electrochemical oxidation and reduction of FHX. However, it should be pointed out that the *y*-intercept deviates from zero, which contrasts with the expectation in a purely diffusion-controlled process where the intercept is typically zero [42].

To determine the diffusion coefficient of FHX, the chronoamperometric procedure previously applied to hydrazine in our previous study [31] was used. Chronoamperometric current–time curves of 1.0 mmol L^−1^ FHX at the PGE were recorded by applying a constant potential of + 700 mV vs. Ag│AgCl_(sat. KCl)_ in a pH 2.0 of BRB solution containing 0.1 mol L^−1^ KCl. The plots of *I* (μA) versus *t*^−1/2^ (s^−1/2^) were derived from the Cottrell equation: $I=\frac{n\times F\times ESA\times {{D}_{FHX}}^{1/2}\times {c}_{FHX}}{{\pi }^{1/2}\times {t}^{1/2}}$, where *n* is the number of electrons (*n* = 2 for FHX oxidation), *F* is Faraday’s constant (96,485 C·mol^−1^), ESA is the electroactive surface area of 1 cm of PGE (0.181cm^2^), *D*_*FHX*_ is the diffusion coefficient of FHX (cm^2^ s^−1^), and *c* is the bulk concentration of FHX (1.0 × 10^−6^ mol cm^−3^). The *D*_*FHX*_ was calculated from the slopes of plots as 1.72 × 10^−6^ ± 5.5 × 10^−8^ cm^2^ s^−1^ (*n* = 3). Additionally, the dimensionless electrode kinetic parameter (*K*) was calculated to be 3.02 from the equation: ΔE_p/2_ = − 10 85 ln(K) + 80 [43], where ΔE_p/2_ was determined to be 65 mV from SW voltammograms of 1.0 µmol L^−1^ FHX using a frequency of 15 Hz, amplitude of 50 mV, and step potential of 4 mV. The standard rate constant of electron transfer (*k*_*s*_) was calculated as 3.96 × 10^−3^ cm s^−1^ using the equation: *K* = (*k*_*s*_ × *f* × *D*)^1/2^ [43].

### Differential pulse voltammetric determination of FHX at the PGE

To conduct the electrochemical determination of FHX, both oxidation and reduction peaks were monitored using the DPV technique. To optimize the procedure, parameters such as pH, pulse amplitude (ΔE_p_), pulse time (*t*_*p*_), step potential (ΔE_s_), and scan rate (*ʋ*) were adjusted to achieve the best voltammetric response.

Optimization studies were initiated by recording the DPVs of 0.25 µmol L^−1^ FHX in 0.1 mol L^−1^ H_2_SO_4_ and in BRB solutions containing 0.1 mol L^−1^ KCl solution with pH values ranging from 2.0 to 10.0 in both anodic and cathodic directions. The recorded DPVs (Fig. S4 and Fig. S5) revealed that both anodic and cathodic peak potentials shifted in more negative directions with increasing pH value, with the highest peak current values being observed in a highly acidic supporting electrolyte (pH 2.0 or 0.1 mol L^−1^ H_2_SO_4_). These findings are consistent with those obtained from CV studies depicted in Fig. 2 and Fig. S2. Therefore, a pH 2.0 of BRB solution containing 0.1 mol L^−1^ KCl solution was adopted as a supporting electrolyte for subsequent studies.

Furthermore, the DPV parameters were optimized by recording DPVs in a BRB solution at pH 2.0 with 0.25 µmol L^−1^ FHX in both anodic and cathodic directions. The parameters were optimized within the following ranges: ΔE_p_ of 50–300 mV, *t*_*p*_ of 1–10 ms, ΔE_s_ of 3–10 mV, and *ʋ* of 25–200 mV s^−1^. Graphs of peak current vs. each parameter studied (Figs. S6–S9 for anodic peak and Figs. S10–S13 for cathodic peak) indicate that most suitable (optimized) parameters values are ΔE_p_ of 180 mV, *t*_*p*_ of 2 ms, ΔE_s_ of 10 mV, and *ʋ* of 50 mV s^−1^ for the anodic peak, and ΔE_p_ of 200 mV, *t*_*p*_ of 2 ms, ΔE_s_ of 15 mV, and *ʋ* of 50 mV s^−1^ for the cathodic peak.

**Fig. 4:**
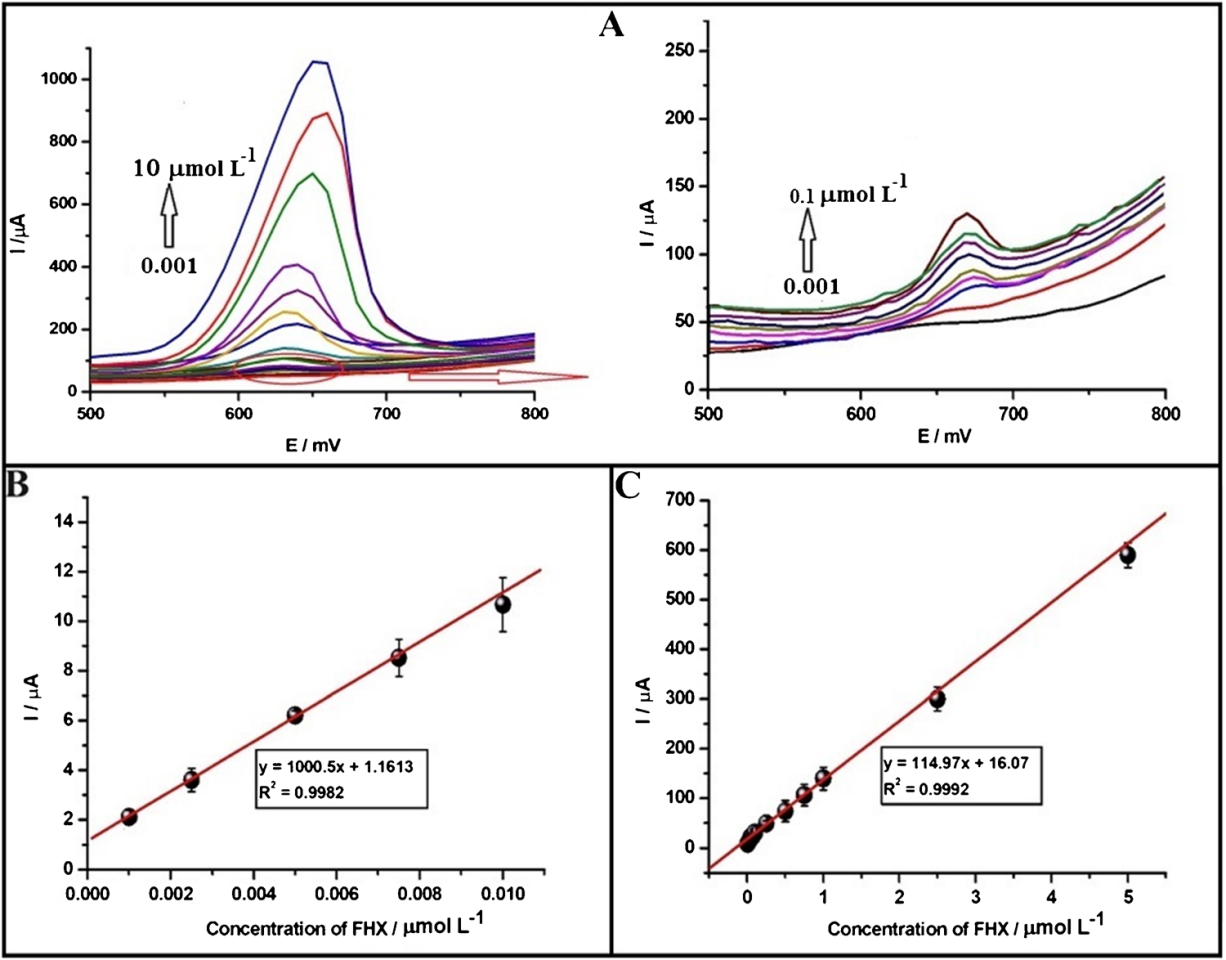
**A** DPVs of FHX showing concentration-dependent (from 0.001 to 10 µmol L^−1^) responses scanned towards the anodic region using the PGE in pH 2.0 of BRB solution containing 0.1 mol L^−1^ KCl solution. The magnified version of DPVs for the range between 0.001 and 0.1 µmol L^−1^ is provided on the right side. Linear calibration curves in the range (**B**) from 0.001 to 0.01 µmol L^−1^ and (**C**) from 0.01 to 5.0 µmol L^−1^

**Fig. 5:**
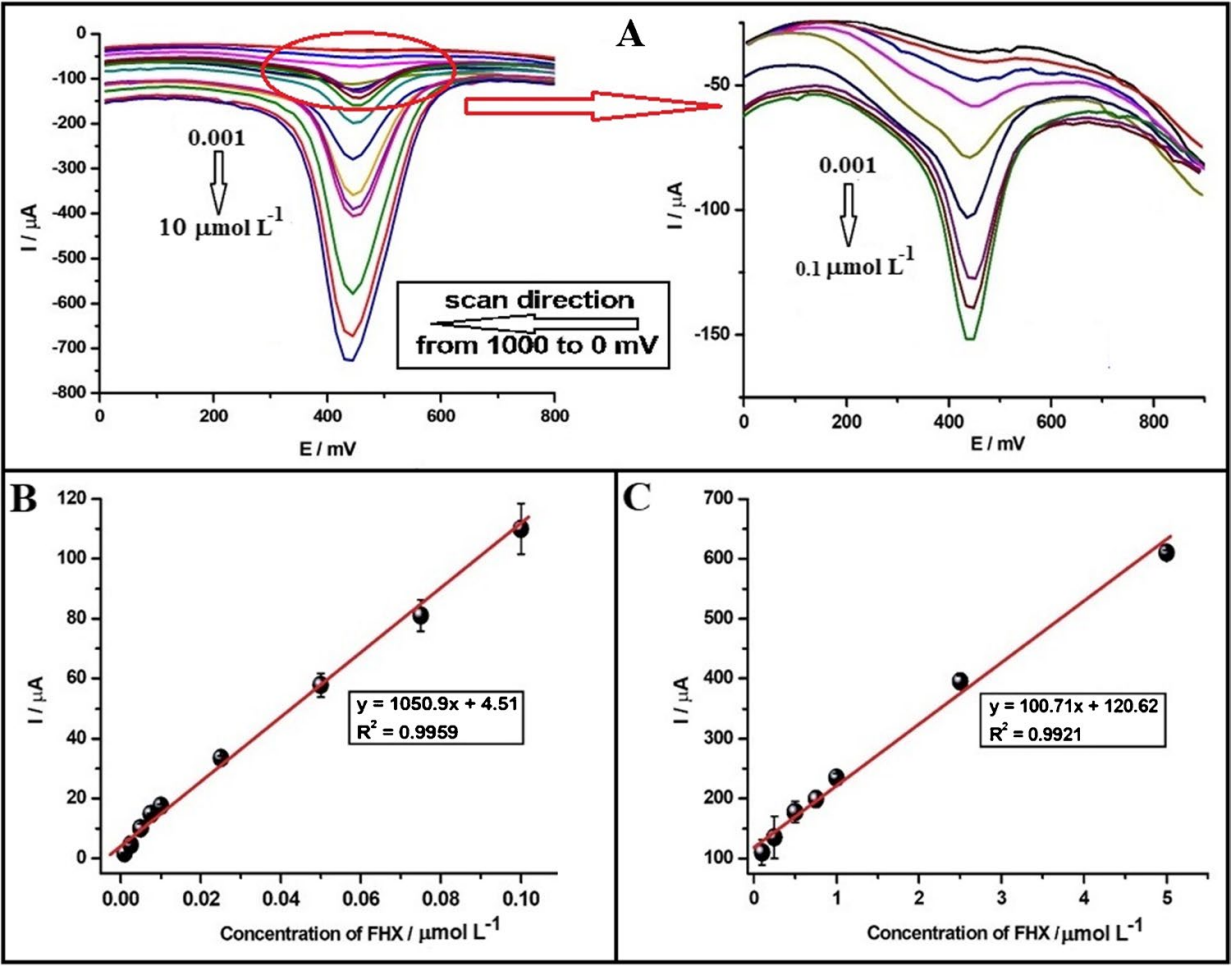
**A** DPVs of FHX showing concentration-dependent (from 0.001 to 10 µmol L^−1^) responses scanned towards the cathodic region using the PGE in pH 2.0 of BRB solution containing 0.10 M KCl solution. The magnified version of DPVs for the range between 0.001 and 0.1 µmol L^−1^ is given on the right side. Linear calibration curves in the range from (**B**) 0.001 to 0.1 µmol L^−1^ and (**C**) from 0.1 to 5.0 µmol L^−^^1^

Under these optimized conditions, analytical performance studies were carried out by recording DPVs in both anodic and cathodic directions across a concentration range of FHX from 0.001 to 10.0 µmol L^−1^ using the PGE (Figs. 4A and 5A). Dynamic calibration curves of both anodic and cathodic peaks indicated a limit of linearity at 5.0 µM (Fig. S14). The calibration curves presented in Figs. 4 and 5 (B and C) demonstrate proportional increase in both anodic and cathodic peak currents with increasing FHX concentration, characterized by two linear segments. For the anodic peak, the first linear segment span from 0.001 to 0.01 µmol L^−1^ with the equation: *I*_*p*_ (µA) = 1000.5*c* (µmol L^−1^) + 1.16 (*R*^2^ = 0.9982), and the second linear segment ranges from 0.01 to 5.0 µmol L^−1^ with the equation: *I*_p_ (µA) = 114.97*c* (µmol L^−1^) + 16.07 (*R*^2^ = 0.9992) (Fig. 4B and C). Regarding the cathodic peak, the following linear equations were obtained: *I*_p_ (µA) = 1050.9*c* (µmol L^−1^) + 4.51 (*R*^2^ = 0.9959) within the concentration range of 0.001 to 0.1 µmol L^−1^ and *I*_p_ (µA) = 105.71*c* (µmol L^−1^) + 120.62 (*R*^2^ = 0.9921) within the concentration range of 0.1 to 5.0 µmol L^−1^ (Fig. 5B and C). The calibration curves revealed that the slopes for the anodic (1000.5 µA L µmol^−1^) and cathodic (1050.9 µA L µmol^−1^) peaks were very close, indicating comparable sensitivities. However, the first linear range for the cathodic peak was found to be wider, and the second linear range was narrower compared to that obtained for the anodic peak. Additionally, the limit of detection (LOD) and limit of quantification (LOQ) values for FHX at the PGE were calculated using the Eqs. 3 × (*S*_*d*_/slope) for LOD and 10 × (*S*_*d*_/slope) for LOQ, where the *S*_*d*_ represents the standard deviation of the smallest FHX concentration that gives an observable signal considered blank. The LOD and LOQ values were determined to be 0.34 nmol L^−1^ and 1.13 nmol L^−1^, respectively, for the anodic peak, and 0.32 nmol L^−1^ and 1.07 nmol L^−1^, respectively, for the cathodic peak.

The precision of the method was evaluated by recording DPVs for two different concentrations of FHX, each repeated 5-times in inter-day and intra-day experiments. For the cathodic peak of FHX, the relative standard deviation (RSD) values obtained from inter-day experiments were found to be 3.7% (for 0.01 µmol L^−1^) and 1.5% (for 1.0 µmol L^−1^), whereas from intra-day experiments, the RSD values were 2.4% (for 0.01 µmol L^−1^) and 4.5% (for 1.0 µmol L^−1^). Regarding the oxidation peak of FHX, RSD values were found to be 2.2% (for 0.01 µmol L^−1^) and 3.4% (for 1.0 µmol L^−1^) for inter-day precision, and 4.2% (for 0.01 µmol L^−1^) and 4.5% (for 1.0 µmol L^−1^) for intra-day precision. The obtained RSD values below 5% for both concentrations demonstrate the good precision of the method using PGE.

Based on our literature review, only a few studies have focused on the voltammetric determination of the important fungicide FHX [27, 28]. In the earlier reports by our co-authors Brycht and Skrzypek, the responses of FHX on other carbon-based electrodes than the PGE were examined in detail and compared (the best results were obtained on the GCPE [27] and APT-BDDE [28]). The analytical performance of the PGE was then compared with two previous electrochemical studies, as well as with chromatographic and ELISA methods. As shown in Table 1, the PGE demonstrates higher sensitivity and lower LOD values compared to other carbon-based electrodes in the voltammetric determination of FHX. Furthermore, our study investigates both oxidation and reduction processes of FHX using the PGE, whereas previous studies on the GCPE and APT-BDDE focused solely on the oxidation of FHX. Additionally, the PGE offers advantages such as being single-use, readily available for purchase, featuring a renewable surface, and eliminating the need for surface pretreatment steps required by other carbon-based electrodes. Moreover, the LOD values obtained from the voltammetric method using the PGE—an inexpensive, readily available, and single-use electrode without modification—were found to be lower than those from chromatographic methods and competitive with ELISA methods. However, chromatographic and ELISA procedures are time-consuming and expensive, and require complex sample preparation steps. Thus, the significant improvement in sensitivity of the low cost, disposability, and simplicity of the electrochemical method using the PGE highlights the novelty of this study.

**Table 1:** Comparison of the analytical performance parameters of PGE to those of previously published FHX determination methods

Method	Procedure	Sensitivity (µA L µmol^−1^)	LOD	Linear range	Sample	Ref
Chromatographic	GNP-ICS using LC/MS	-	0.79 ng mL^**−**1^ (2.6 × 10^**−**3^ µmol L^**−**1^)	0–100 ng mL^**−**1^ (0–0.33 µmol L ^**−**1^)	Cucumber, and grape	[19]
	GC-ECD *GC-NPD	-	0.05 mg L^**−**1^ (0.17 µmol L^**−**1^) *0.14 mg L^**−**1^ (0.46 µmol L^**−**1^)	0.025–10 mg L^**−**1^ (0.083–33.1 µmol L^**−**1^) *0.25–10 mg L^**−**1^ (~ 0.83–33.1 µmol L^**−**1^)	Grape, tomato, and wine	[20]
	GC-NPD *HPLC-UVD (200 nm) sing C-18 or Supelpak-2 for sampling	-	0.2 ng µL^**−**1^ (~ 0.66 µmol L^**−**1^) *0.02 µg mL^**−**1^ (~ 0.066 µmol L^**−**1^)	-	Greenhouse air Tomato crop	[21]
	HPLC-UVD (210 nm)		0.04 ppm (~ 0.13 µmol L^**−**1^)	0.2–0.5 µg mL^**−**1^ (~ 0.66–1.65 µmol L^**−**1^)	Pepper fruits	[22]
	LC–MS/MS		0.009 ppm (~ 0.030 µmol L^**−**1^)	-	Caneberry, blueberry, and pomegranate	[23]
ELISA	ic-ELISA		0.04 µg L^**−**1^ (~ 1.32 × 10^**−**4^ µmol L^**−**1^)	-	Grape must, kiwifruit, and strawberry	[24]
	c-ELISA		4 ng L^**−**1^ (~ 1.32 × 10^**−**5^ µmol L^**−**1^)	-	Cucumber, and tomato	[25]
	dc-ELISA		0.03 µg L^**−**1^ (~ 9.93 × 10^**−**5^ µmol L^**−**1^)	-	Must, and wine	[26]
Electrochemical	GCPE-SWV/0.1 mol L^**−**1^ BRB, pH 4.0 + 0.65 V (oxidation)	0.176	1.32 µmol L^**−**1^	3.96–49.50 µmol L^**−**1^	Berries, and wine grapes	[27]
	APT-BDDE-SWV/0.04 mol L^**−**1^ BRB, pH 2.0 + 1.30 V(oxidation)	0.0265	0.821 µmol L^**−**1^	3.0–100.0 µmol L^**−**1^	Blueberries	[28]
	PGE DPV/0.04 mol L^**−**1^ BRB, pH 2.0 + 0.65 V (oxidation) + 0.45 V(reduction)	1000.5 and 115.0 1050.9 and 100.7	3.4 × 10^–4^ µmol L^**−**1^ 3.2 × 10^–4^ µmol L^**−**1^	10^**−**3^–10^**−**2^ and 10^**−**2^–5.0 µmol L^**−**1^ 10^**−**3^–0.1 and 0.1–5.0 µmol L^**−**1^	Water, and soil	This work

*GNP-ICS*, gold nanoparticle-immunochromatographic strip; *LC/MS*, liquid chromatography/mass spectrometry; *GC-ECD*, gas chromatography-electron capture detector; *GC-NPD*, gas chromatography-nitrogen phosphorous detector; *HPLC-UVD*, high-performance liquid chromatography with ultraviolet detector; *ic*-, *c*,- or *dc-ELISA*, indirect competitive, competitive, or direct competitive enzyme-linked immunosorbent assay; *GCPE*, glassy carbon paste electrode; *APT-BDDE*, anodically pretreated boron-doped diamond electrode; *PGE*, pencil graphite electrode; *SWV*, square-wave voltammetry; *DPV*, differential pulse voltammetry; *BRB*, Britton–Robinson buffer

### Selectivity studies

To assess the interference effects of various pesticides and anionic species on the anodic and cathodic responses of FHX, DPVs of the PGE were recorded under optimized conditions in the presence of an analyte-to-interfering compound at different concentration ratios ranging from 1:1 to 1:500. Peak currents were evaluated from recorded DPVs in the absence and presence of interfering compounds at each studied ratio, and percentage changes were calculated. Tolerance limits were determined based on 10% change criterion. Anionic and cationic species (Na^+^, K^+^, Ca^2+^, Cu^2+^, Mn^2+^, Mg^2+^, Zn^2+^, Co^2+^, SO_4_^2−^, Cl^−^, and NO_3_^−^) studied at the same analyte-to-interference ratio (1:500), atrazine and carbendazim at a 1:5 ratio, monolinuron at the 1:1 ratio, and trifluralin at the 1:10 ratio did not exhibit interference on both reduction and oxidation of FHX (Table S2). The effects of electroactive molecules, such as ascorbic acid (AA), uric acid (UA), and dopamine (DA), on the anodic and cathodic peak currents of FHX were also investigated. The results revealed that AA did not interfere with the anodic or cathodic peaks of FHX at a 1:500 ratio. UA did not affect the cathodic and anodic peaks of FHX at a 1:100 ratio, however, at higher concentrations (over 100 µM, corresponding to a 1:100 ratio), it caused broadening of the oxidation peak. DA did not interfere with the anodic peak of FHX at 1:500, but at a 1:10 ratio, its reduction peak overlapped with that of FHX. These studies demonstrate that voltammetric determination of FHX based on both oxidation and reduction offers significant advantages. When an interferent affects the oxidation of FHX, its reduction peak can be used, and vice versa, allowing flexibility in analysis. Therefore, considering both peaks of FHX is crucial for minimizing possible interference from other compounds. These findings indicate that the voltammetric determination of FHX using the PGE can be selectively performed for both oxidation and reduction processes in the presence of other pesticides, electroactive compounds such as AA, DA and UA, and ionic species at the defined analyte-to-interfering compound ratios.

### Application to real samples

The proposed procedure was used to quantify FHX in various real samples, including irrigation water, tap water, and soil. For this purpose, DPVs of samples spiked with known FHX concentrations were recorded under optimized conditions. The standard addition method was employed for all spiked samples, with DPVs recorded after each standard addition. By evaluating the anodic and cathodic peak currents from the DPVs obtained for each sample, recovery values for FHX spiked in the samples were calculated (Table 2), and they were close to 100%. These results indicate that the voltammetric determination of FHX using the PGE, based on both oxidation and reduction peaks, can be effectively applied to water and soil samples with high accuracy.

**Table 2:** Results obtained from the real sample analysis based on both reduction and oxidation signals of FHX at the PGE (*n* = 3)

Sample	Spiked (µmol L^−1^)	Reduction		Oxidation	
		Found (µmol L^−1^)	Recovery (%)	Found (µmol L^−1^)	Recovery (%)
Tap water	0	-	-	-	-
	5	5.6 ± 0.3	112.0 ± 2.4	5.25 ± 0.2	105.0 ± 2.2
	10	10.8 ± 0.4	108.0 ± 2.2	11.1 ± 0.1	111.0 ± 1.4
	15	15.9 ± 0.4	106.0 ± 2.8	15.45 ± 0.2	103.0 ± 1.4
Irrigational water	0	-	-	-	-
	5	5.6 ± 0.1	111.0 ± 1.4	5.3 ± 0.1	105.0 ± 1.4
	10	10.9 ± 0.1	109.0 ± 1.4	9.7 ± 0.1	97.0 ± 1.4
	15	15.3 ± 0.4	102.0 ± 2.3	15.2 ± 0.2	101.3 ± 1.2
Soil	0	-	-	-	-
	10	10.8 ± 0.2	108.0 ± 1.8	9.8 ± 0.3	98.0 ± 2.8
	20	22.1 ± 1.0	110.5 ± 2.7	21.6 ± 3.3	108.0 ± 3.3
	40	39.9 ± 0.9	99.8 ± 2.5	43.9 ± 4.7	109.8 ± 2.6

## Conclusion

In this study, a disposable and cost-effective PGE was utilized as a robust sensing platform for the straightforward, reliable, sensitive, and selective determination of the fungicide FHX, an essential and widely used agricultural chemical. The determination of FHX was based on both its oxidation peak at approximately + 0.65 V and its reduction at + 0.44 V vs. Ag│AgCl_(sat. KCl)_, highlighting the versatility and effectiveness of the method. DP voltammetric results showed two linear ranges: 0.001–0.01 µmol L^−1^ and 0.01–5.0 µmol L^−1^for the anodic peak, and 0.001–0.1 µmol L^−1^ and 0.1–5.0 µmol L^−1^ for the cathodic peak. The limit of detection values were estimated to be 0.34 nmol L^−1^ and 0.32 nmol L^−1^ for the anodic and cathodic peaks, respectively. These results indicate that the bare PGE exhibits excellent sensitivity for the detection of FHX. Comparative analysis with other voltammetric approaches using alternative carbon-based electrodes highlighted the superior electroanalytical performance of the PGE, particularly in practical pesticide analysis. The PGE offers distinct advantages over other electrodes, including rapid analysis, affordability, disposability, high sensitivity and selectivity, simplicity, and elimination of the need for surface pretreatment. Furthermore, interference studies with various pesticides and ionic species demonstrated minimal impact on FHX determination, enhancing the method’s utility for real sample analysis, as evidenced by successful applications in real samples (irrigation water, tap water, and soil). The standard addition method employed for recovery assessment yielded satisfactory results, with recovery values close to 100%, indicating high accuracy and reliability of the developed method in real-world scenarios.

Overall, the proposed voltammetric approach offers a promising tool for FHX determination in environmental samples, providing valuable insights for pesticide monitoring and environmental assessment. Consequently, the PGE emerges as an economical and promising analytical tool, offering effective solutions not only for environmental analyses but also for applications in pharmaceutical, clinical, and food industries.

## Supplementary Information

Below is the link to the electronic supplementary material.

Supplementary file1 (DOCX 3.19 MB)

## Data Availability

No datasets were generated or analysed during the current study.
