# Supplementary material for: Highly sensitive voltammetric determination of the fungicide fenhexamid using a cost-effective and disposable pencil graphite electrode
Source: Mikrochim Acta. 2024 Nov 29;191(12):773. doi: 10.1007/s00604-024-06804-4 (PMC11607022; doi:10.1007/s00604-024-06804-4)
Supplement: Supplementary file 1 — Supplementary file1 (DOCX 3.19 MB) [file 604_2024_6804_MOESM1_ESM.docx]

**SUPPLEMENTARY MATERIALS**

**Highly sensitive voltammetric determination of the fungicide fenhexamid using a cost-effective and disposable pencil graphite electrode**

Teslime Erşan^1^, Didem Giray Dilgin^2^, Ayhan Oral^1^, Sławomira Skrzypek^3^, Mariola Brycht*^,3^, Yusuf Dilgin*^,1^

*^1^Çanakkale Onsekiz Mart University, Faculty of Science, Department of Chemistry, Canakkale, Turkey*

*^2^Çanakkale Onsekiz Mart University, Faculty of Education,* Secondary Science and Mathematics Education Department, Çanakkale, Turkey

*^3^University of Lodz, Faculty of Chemistry, Department of Inorganic and Analytical Chemistry, Tamka 12, 91-403 Łódź, Poland*

**
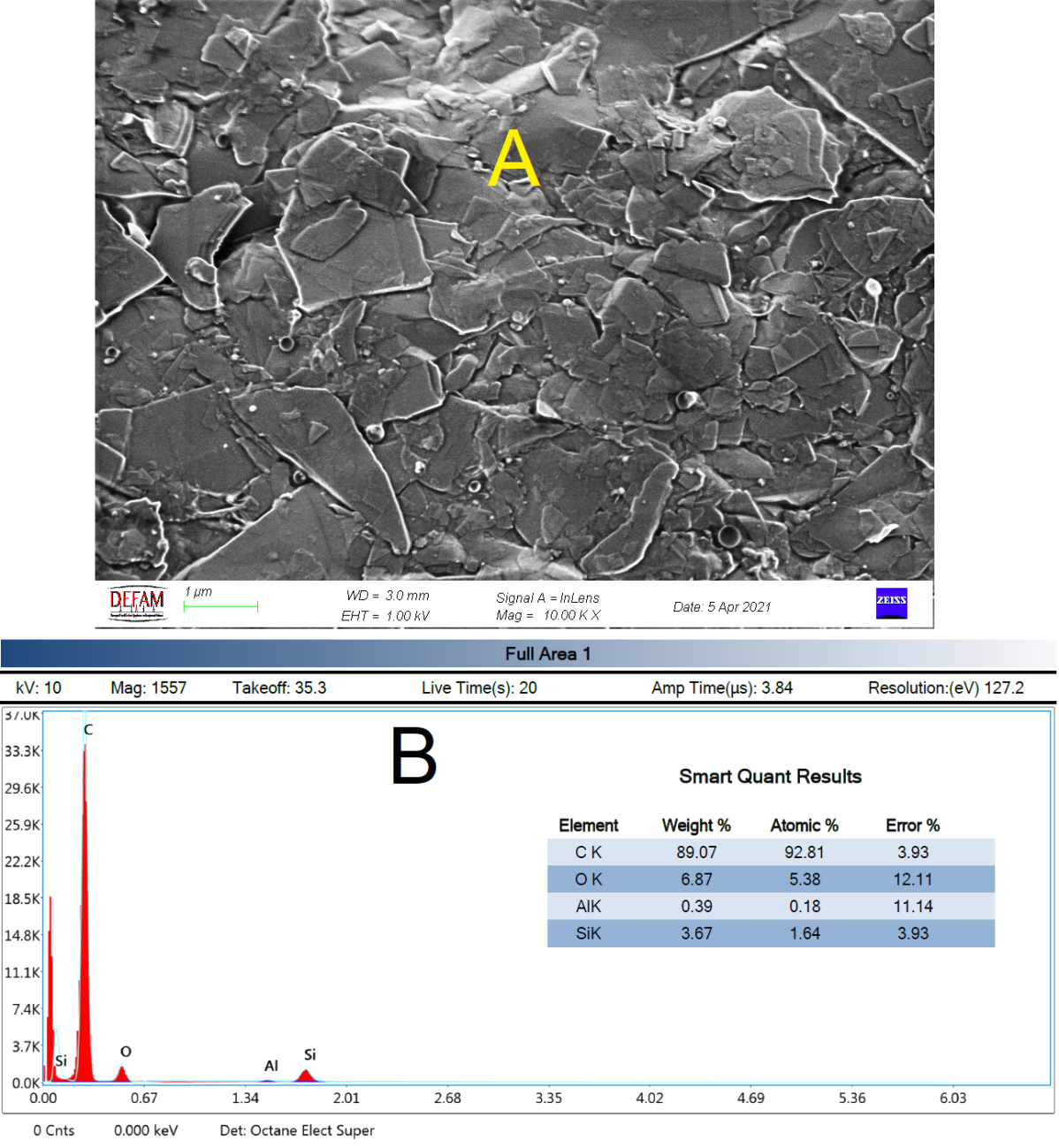
**

**Fig. S1**. FE-SEM image and EDX spectrum of pencil leads. Inset: Elementel composition of pencil leads from the EDX spectrum.

**
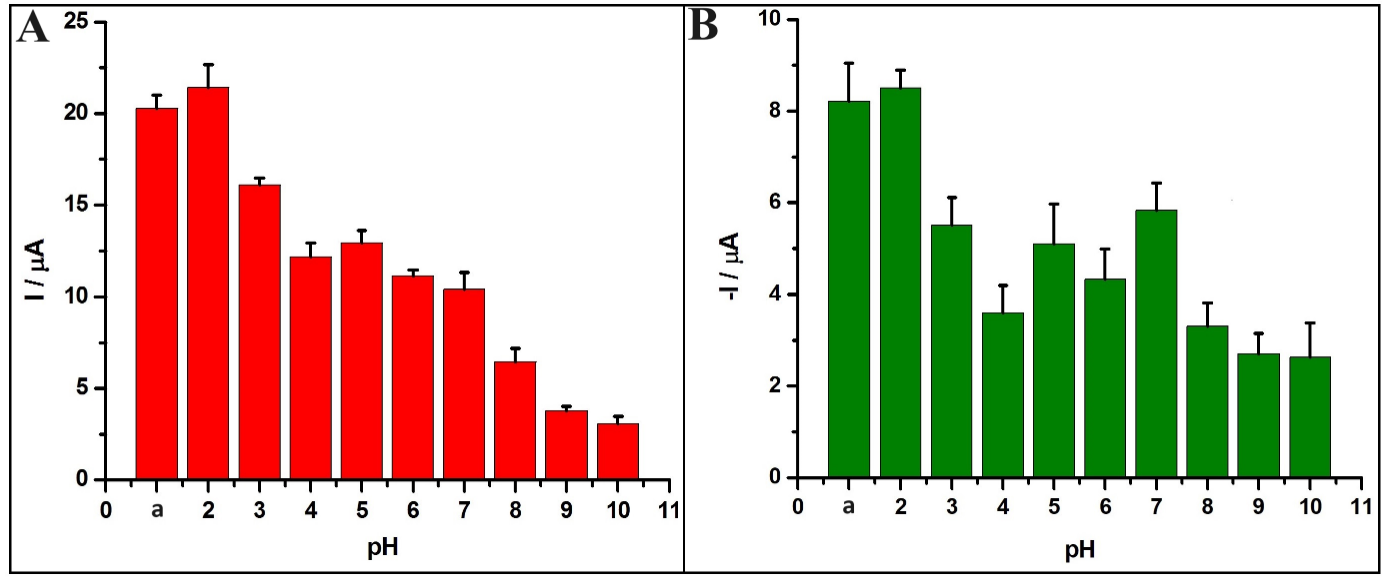
**

**Fig. S2.** Bar graphs illustrating the oxidation **(A)** and reduction **(B)** peak currents vs pH (a: 0.1 mol L^–1^ H_2_SO_4_). Error bars were constructed as standard deviations (n = 3).


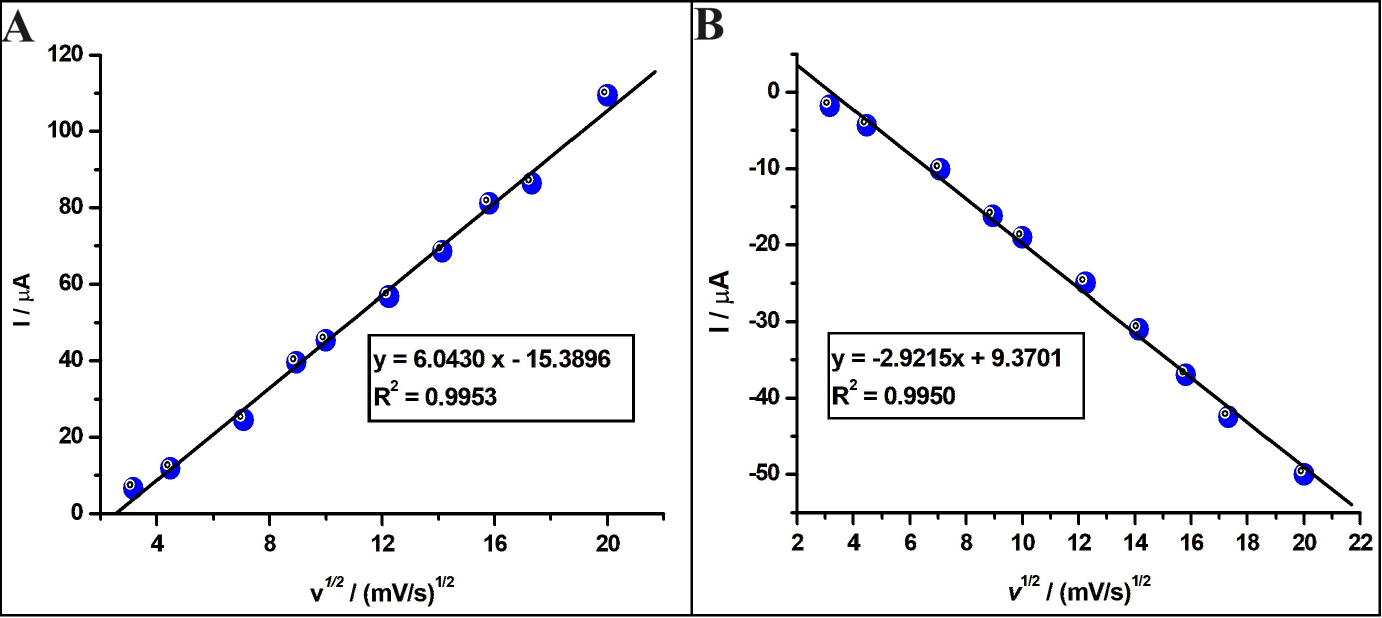


**Fig. S3.** Linear dependencies of currents vs the square root of the scan rate for **(A)** anodic and **(B)** cathodic peaks.


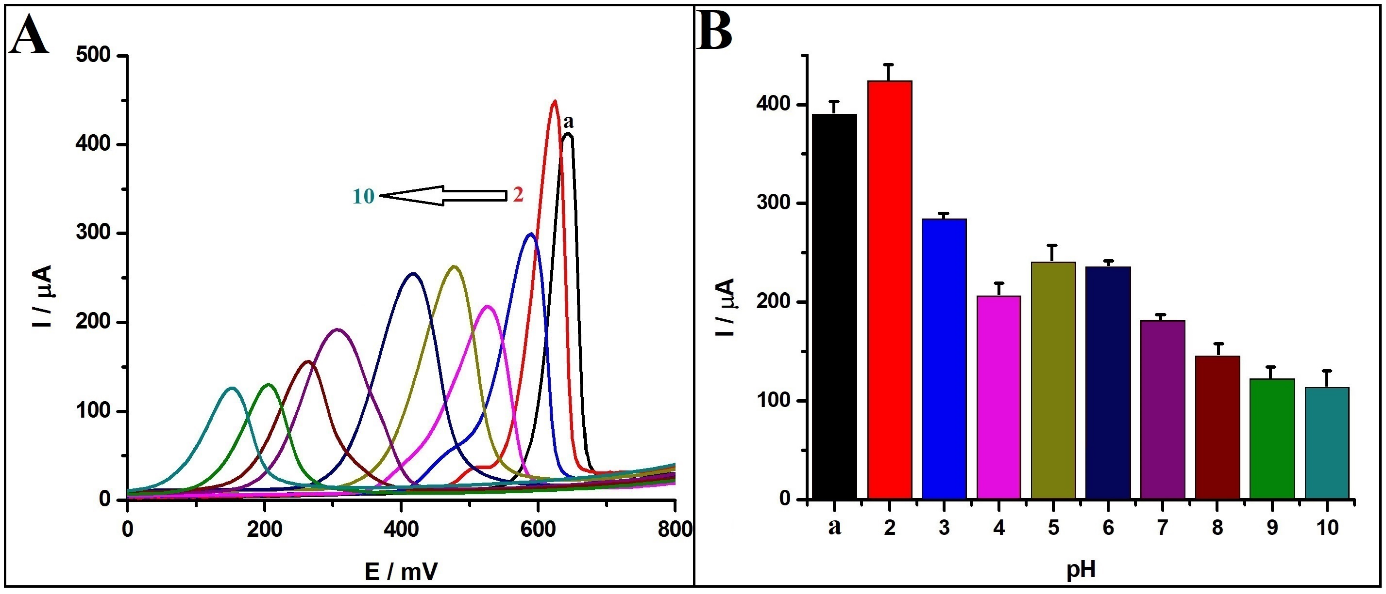


**Fig. S4. (A)** DPVs of 0.25 µmol L^–1^ FHX scanned towards anodic region at the PGE in 0.1 mol L^–1^ H_2_SO_4_ and BRB solutions containing 0.1 mol L^–1^ KCl solution prepared at varying pHs between 2.0 and 10.0 at (ΔE_p_ of 180 mV, t_p_ of 2 ms, ΔE_es_ of 10 mV, and ʋ of 50 mV s^–1^). **(B)** A graph bar of oxidation peak current vs pH (a: 0.1 mol L^–1^ H_2_SO_4_). Error bars were constructed as standard deviations (n = 3).


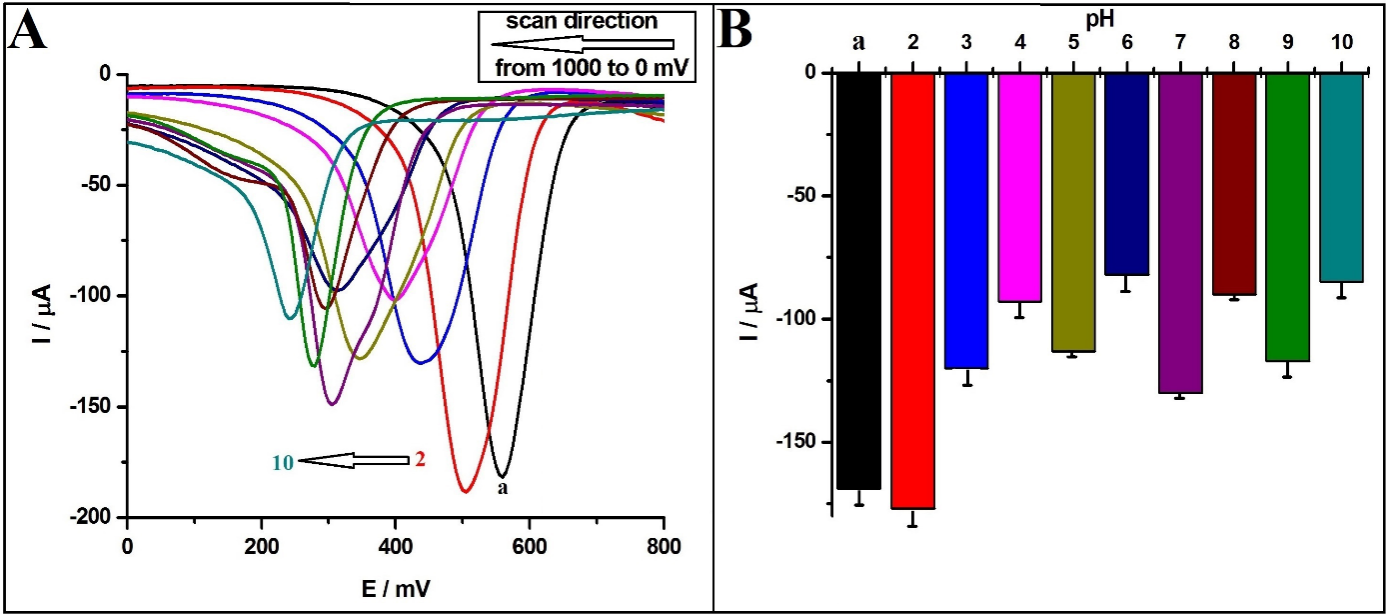


**Fig. S5. (A)** DPVs of 0.25 µmol L^–1^ FHX scanned towards cathodic region at the PGE in 0.1 mol L^–1^ H_2_SO_4_ and BRB solutions containing 0.1 mol L^–1^ KCl solution prepared at varying pH values between 2.0 and 10.0 at a scan rate of 50 mV s^–1^ (ΔE_p_ of 200 mV, t_p_ of 2 ms, ΔE_es_ of 15 mV, and ʋ of 50 mV s^–1^). (**B)** A graph bar of reduction peak current vs pH (a: 0.1 mol L^–1^H_2_SO_4_). Error bars were constructed as standard deviations (n = 3).


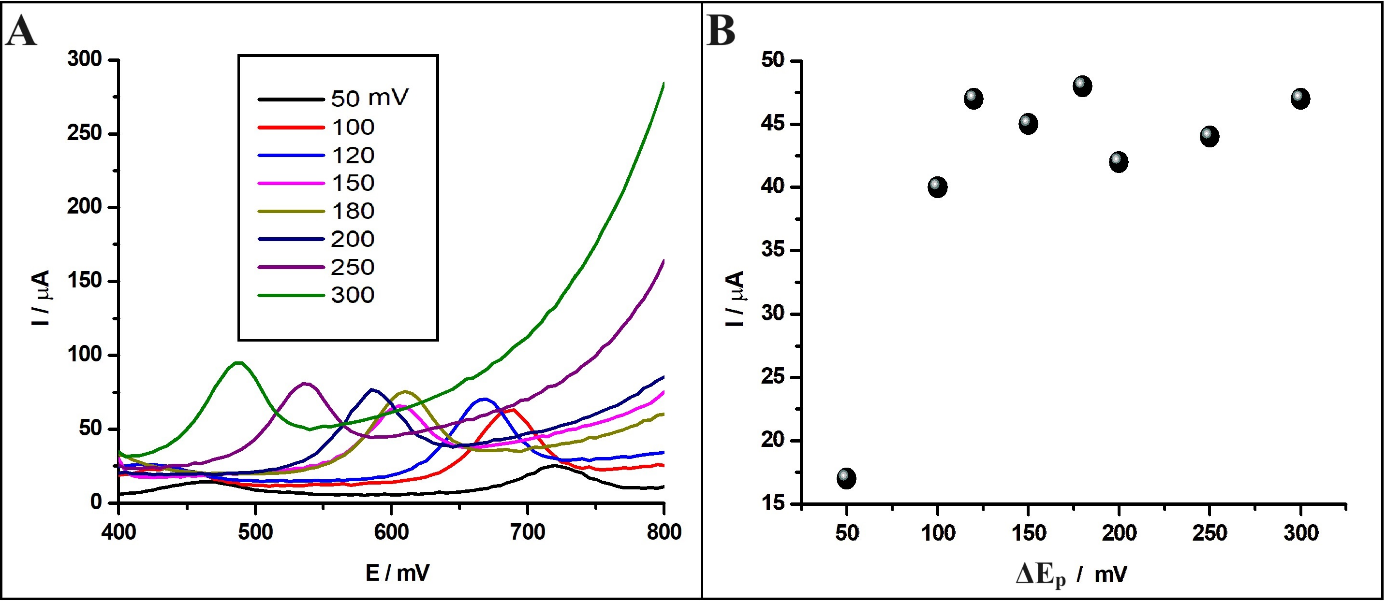


**Fig. S6. (A)** DPVs of 0.25 µmol L^–1^ FHX scanned towards anodic region at the PGE in pH 2.0 of BRB solutions containing 0.1 mol L^–1^ KCl solution prepared at varying pulse amplitude (t_p_ of 2 ms, ΔE_s_ of 10 mV, and ʋ of 50 mV s^–1^). **(B)** Relationship between oxidation peak current and pulse amplitude.


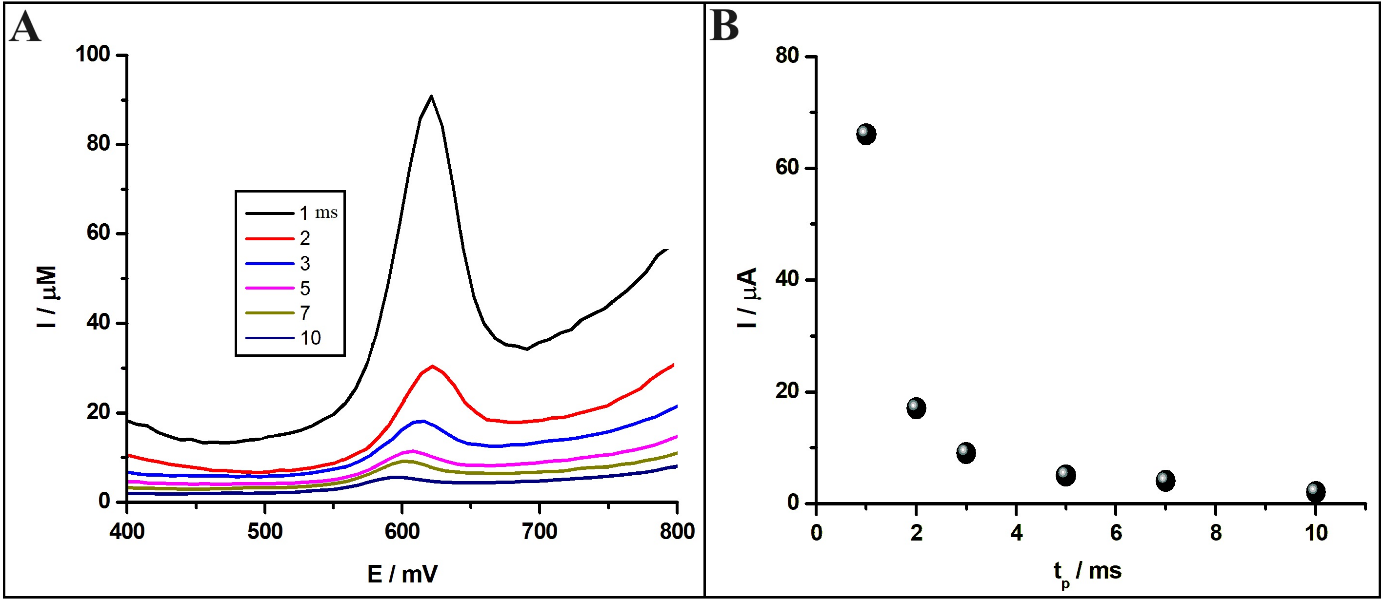


**Fig. S7. (A)** DPVs of 0.25 µmol L^–1^ FHX scanned towards anodic region at the PGE in pH 2.0 of BRB solution containing 0.1 mol L^–1^ KCl solution prepared at varying pulse time (ΔE_p_ of 180 mV, ΔE_s_ of 10 mV, and ʋ of 50 mV s^–1^) **(B)** Relationship between oxidation peak current and pulse time.


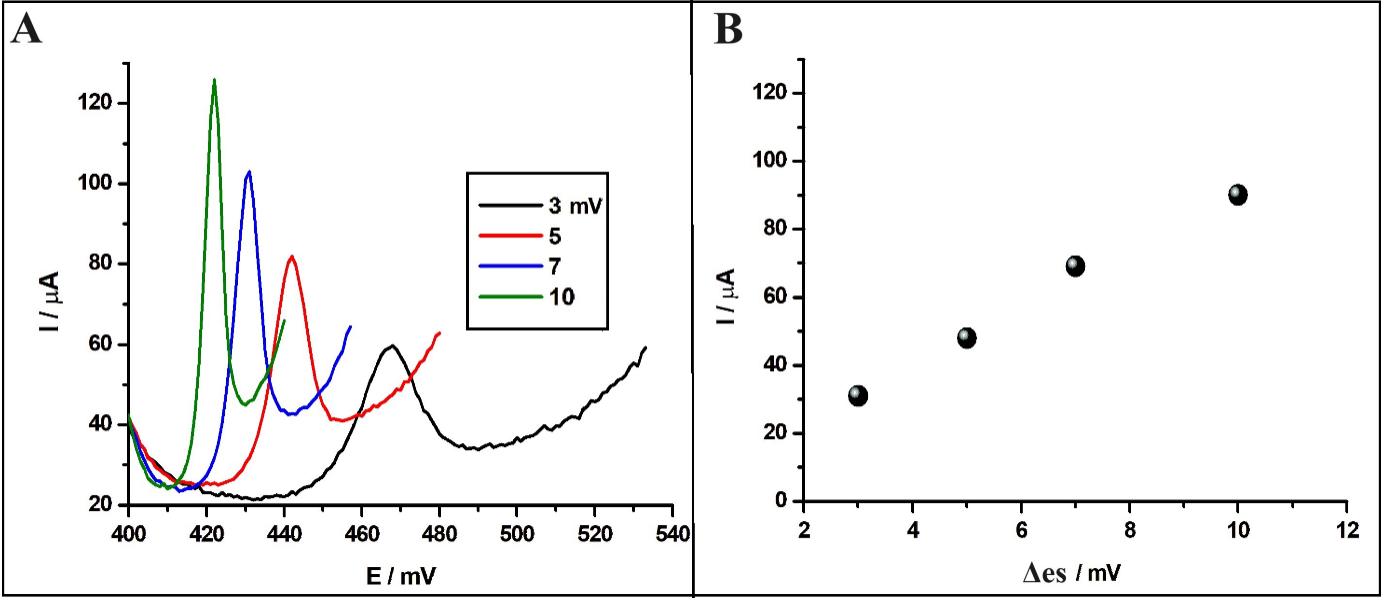


**Fig. S8 (A)** DPVs of 0.25 µmol L^–1^ FHX scanned towards anodic region at the PGE in pH 2.0 of BRB solution containing 0.1 mol L^–1^ KCl solution prepared at varying step potential (ΔE_p_ of 180 mV, t_p_ of 2 ms, and ʋ of 50 mV s^–1^). **(B)** Relationship between oxidation peak current and step potential.


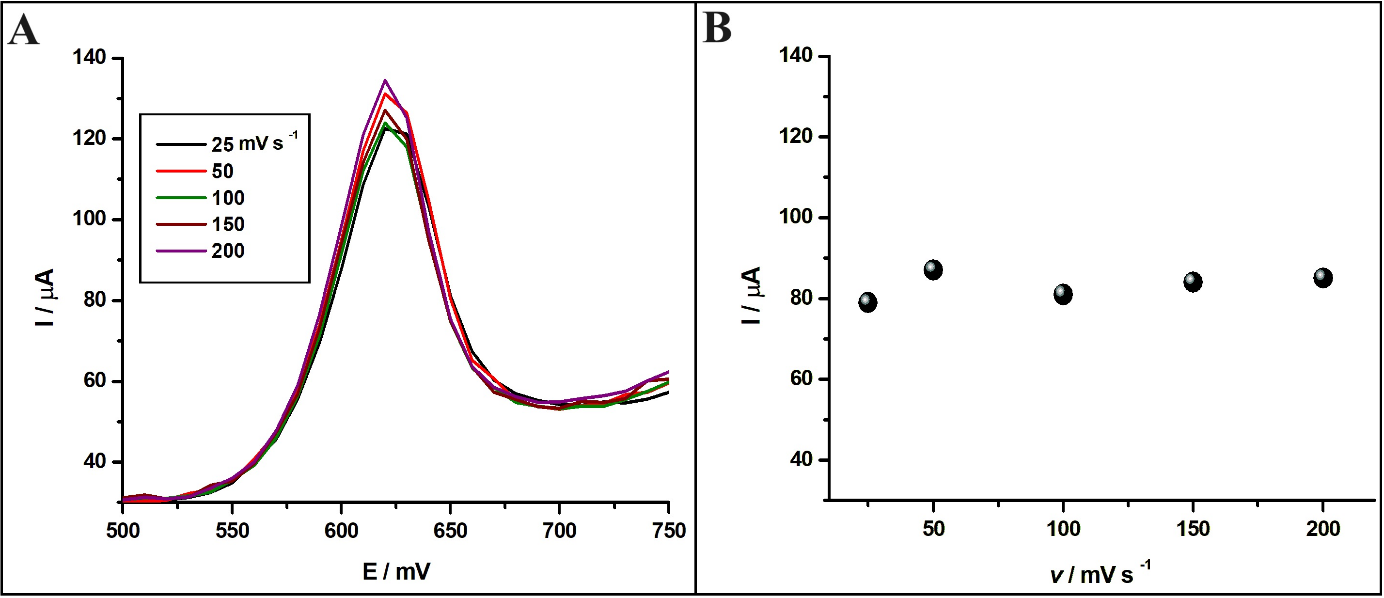


**Fig. S9. (A)** DPVs of 0.25 µmol L^–1^ FHX scanned towards anodic region at the PGE in pH 2.0 of BRB solution containing 0.1 mol L^–1^ KCl solution prepared at varying scan rates (ΔE_p_ of 180 mV, t_p_ of 2 ms, and ΔE_s_ of 10 mV). **(B)** Relationship between oxidation peak current and scan rate.


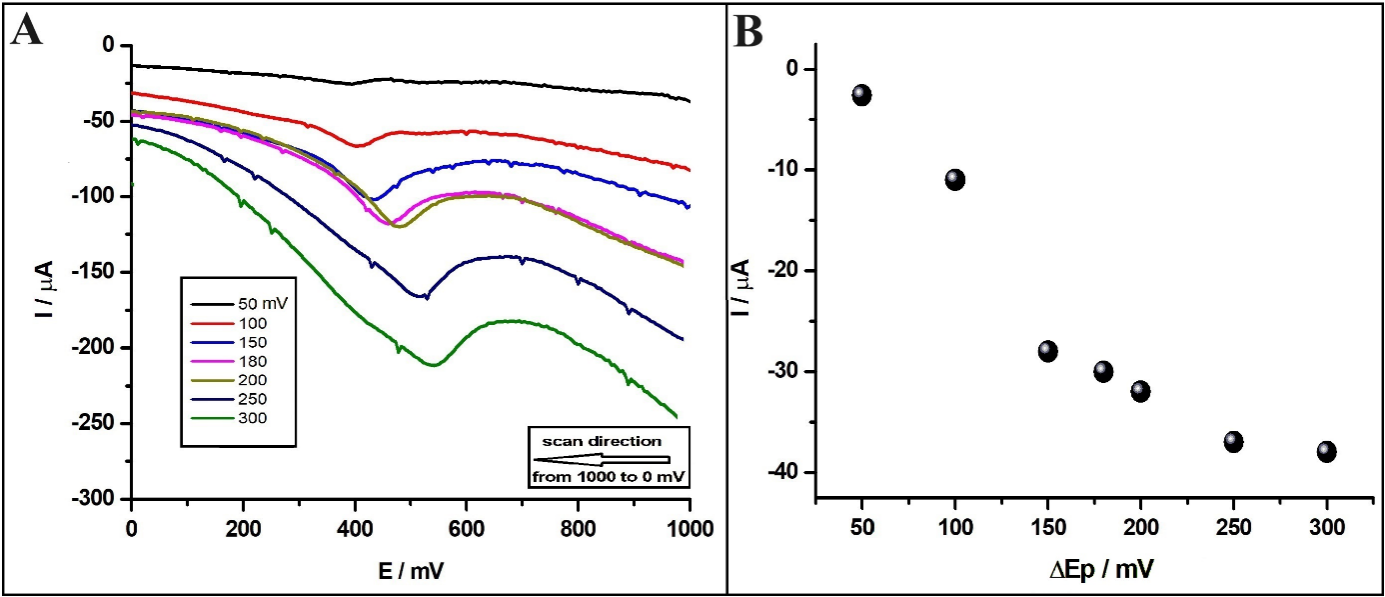


**Fig. S10. (A)** DPVs of 0.25 µmol L^–1^ FHX scanned towards cathodic region at the PGE in pH 2.0 of BRB solution containing 0.1 mol L^–1^ KCl solution prepared at varying pulse amplitude (t_p_ of 2 ms, ΔE_es_ of 15 mV, and ʋ of 50 mV s^–1^). **(B)** Relationship between reduction peak current and pulse amplitude.


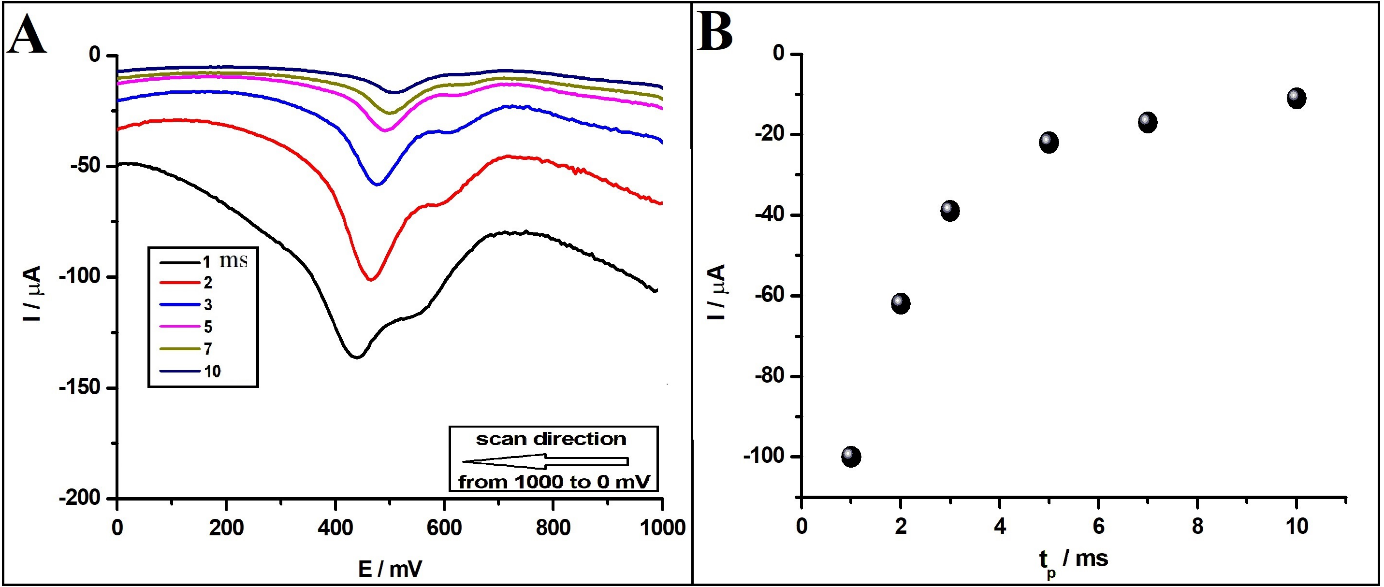


**Fig. S11. (A)** DPVs of 0.25 µmol L^–1^ FHX scanned towards cathodic region at the PGE in pH 2.0 of BRB solution containing 0.1 mol L^–1^ KCl solution prepared at varying pulse time (ΔE_p_ of 200 mV, ΔE_s_ of 15 mV, and ʋ of 50 mV s^–1^). **(B)** Relationship between reduction peak current and pulse time.


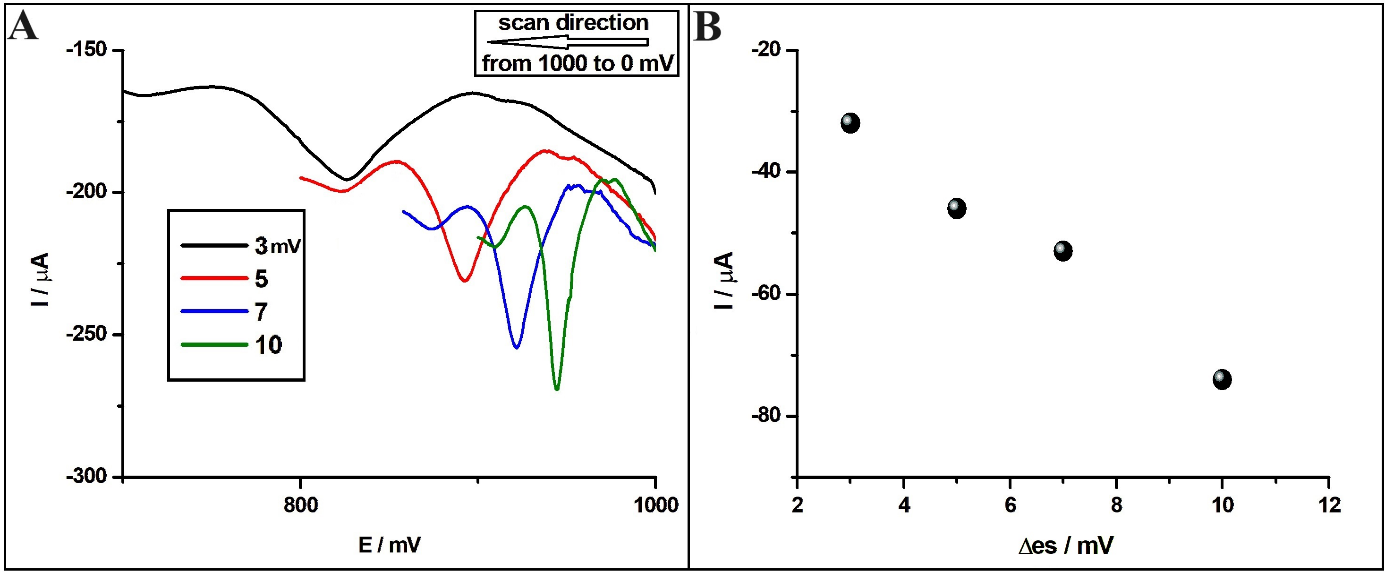


**Fig. S12. (A)** DPVs of 0.25 µmol L^–1^ FHX scanned towards cathodic region at the PGE in pH 2.0 of BRB solution containing 0.1 mol L^–1^ KCl solution prepared at varying step potential (ΔE_p_ of 200 mV, t_p_ of 2 ms, and ʋ of 50 mV s^–1^). **(B)** Relationship between reduction peak current and step potential.


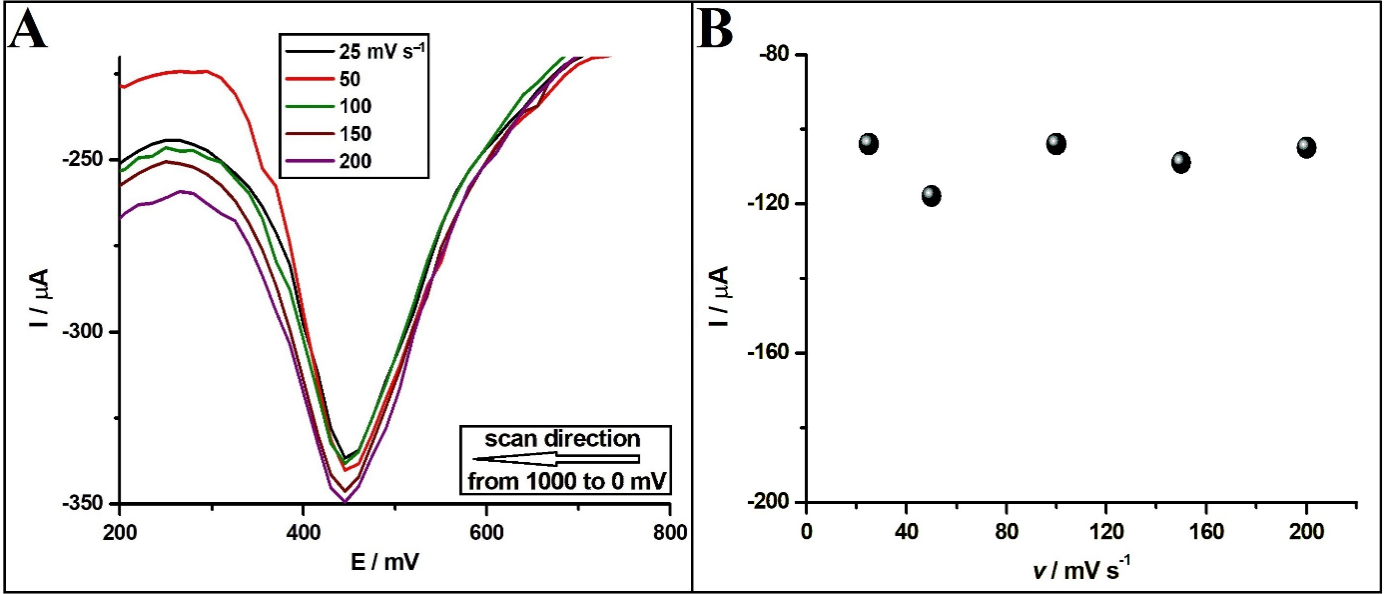


**Fig. S13. (A)** DPVs of 0.25 µmol L^–1^ FHX scanned towards cathodic region at the PGE in pH 2.0 of BRB solution containing 0.1 mol L^–1^ KCl solution prepared at varying scan rates (ΔE_p_ of 200 mV, t_p_ of 2 ms, ΔE_s_ of 15 mV). **(B)** Relationship between reduction peak current and scan rate.


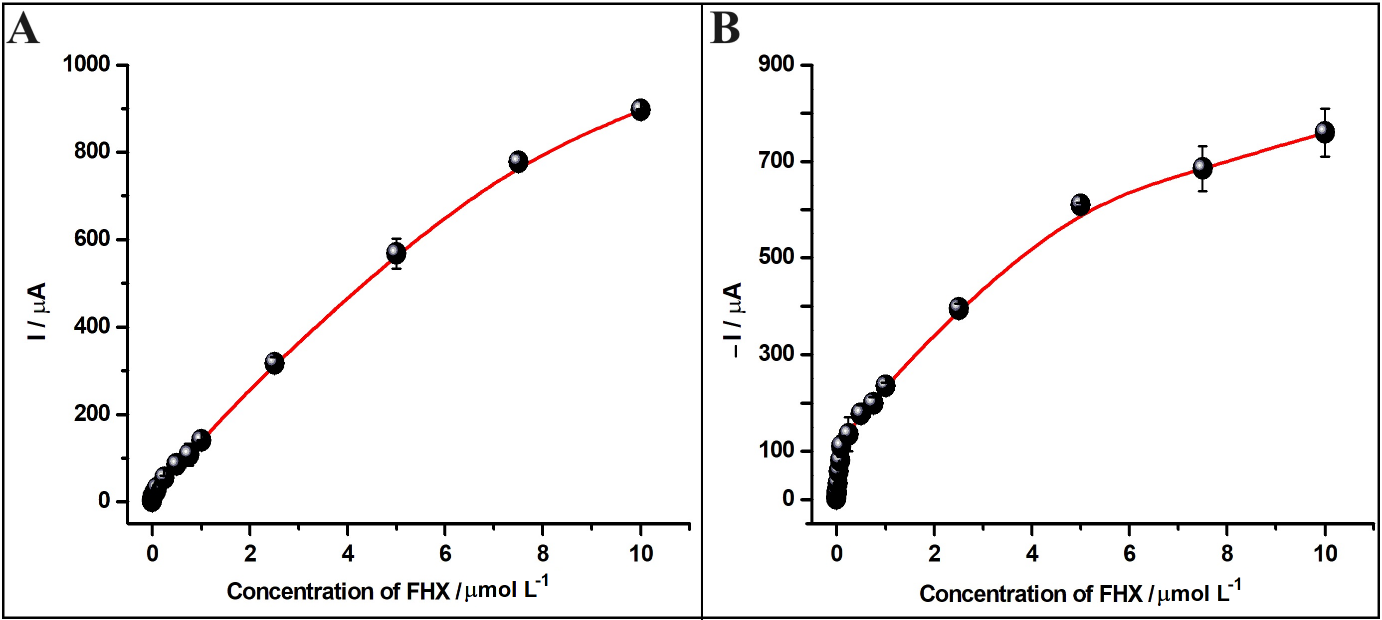


**Fig. S14.** Dependencies of **(A)** anodic and **(B)** cathodic peak currents vs FHX concentration in the range from 0.001 to 10 µmol L^–1^.

**Table S1.** Electroactive surface area, roughness factor, and kinetic parameter of electrodes, obtained from CVs recorded in 0.1 mol L^–1^ [Fe(CN)_6_]^4–/3–^ at scan rate varying from 50 to 500 mV s^–1^ (n=3).

| **Electrodes** | **ESA / cm^2^** | **A_geom_ / cm^2^** | **%Rf [(ESA/A_geom_)×100]** | **k_o_ (cm s^-1)^** | **α_0_** |
| --- | --- | --- | --- | --- | --- |
| PGE | 0.181 ± 0.013 | 0.159 | 113.8 | 1.33×10^–5^ ± 6.11×10^–7^ | 0.52 ± 0.01 |
| CPE | 0.096 ± 0.005 | 0.07 | 137.1 | 3.86×10^–5^ ± 2.05×10^–6^ | 0.79 ± 0.05 |
| SPCE | 0.094 ± 0.001 | 0.126 | 74.6 | 3.64×10^–5^ ± 2.56×10^–6^ | 0.68 ± 0.01 |
| GCE | 0.050 ± 0.002 | 0.07 | 71.42 | 3.71×10^–5^ ± 1.47×10^–6^ | 0.54 ± 0.03 |

**Table S2.** Results obtained from interference study for differential pulse voltammetric determination of FHX at the PGE.

| **Interfering compounds** | **Tolerable molar concentration ratio of analyte:interference / Change in current of FXH (%)** | |
| --- | --- | --- |
|  | **Reduction peak** | **Oxidation peak** |
| MgCl_2_ | 1:500 / 3.19 | 1:500 / 1.02 |
| CuCl_2_ | 1:500 / 2.43 | 1:500 / 6.31 |
| Co(NO_3_)_2_ | 1:500 / 1.86 | 1:500 / 9.09 |
| CaCl_2_ | 1:500 / 9.56 | 1:500 / 6.61 |
| KCl | 1:500 / 8.92 | 1:500 / 8.69 |
| MnSO_4_×H_2_O | 1:500 / 7.62 | 1:500 / 5.22 |
| NaCl | 1:500 / 10.2 | 1:500 / 3.96 |
| ZnCl_2_ | 1:500 / 8.33 | 1:500 / 2.83 |
| Atrazine | 1:5 / -5.78 | 1:5 / -5.17 |
| Monolinuron | 1:1 / -6.66 | 1:1 / -6.31 |
| Trifluralin | 1:10 / -8.98 | 1:10 / -9.09 |
| Carbendazim | 1:5 / -5.19 | 1:5 / -9.58 |
| Ascorbic acid | 1:500 / +6.75 | 1:500 / -1.90 |
| Dopamine | 1:10 / +4.50 | 1:500 / -5.82 |
| Uric acid | 1:100 /-2.17 | 1:100 / +3.53 |
